# BCG Vaccination of Health Care Workers Does Not Reduce SARS-CoV-2 Infections nor Infection Severity or Duration: a Randomized Placebo-Controlled Trial

**DOI:** 10.1128/mbio.00356-23

**Published:** 2023-03-28

**Authors:** Juana Claus, Thijs ten Doesschate, Cheyenne Gumbs, Cornelis H. van Werkhoven, Thomas W. van der Vaart, Axel B. Janssen, Gaby Smits, Rob van Binnendijk, Fiona van der Klis, Debbie van Baarle, Fernanda L. Paganelli, Helen Leavis, Lilly M. Verhagen, Simone A. Joosten, Marc J. M. Bonten, Mihai G. Netea, Janneke H. H. M. van de Wijgert

**Affiliations:** a Julius Center for Health Sciences and Primary Care, University Medical Center Utrecht, Utrecht University, Utrecht, Netherlands; b Department of Internal Medicine, Division of Infectious Diseases, Amsterdam University Medical Center, University of Amsterdam, Amsterdam, Netherlands; c Department of Medical Microbiology, University Medical Center Utrecht, Utrecht University, Utrecht, Netherlands; d National Institute of Public Health and the Environment, Bilthoven, Netherlands; e Department of Medical Microbiology and Infection Prevention, University Medical Center Groningen, Groningen, Netherlands; f Department of Internal Medicine, University Medical Center Utrecht, Utrecht University, Utrecht, Netherlands; g Department of Pediatric Infectious Diseases and Immunology, Radboud Center for Infectious Diseases, Radboud University Medical Center, Nijmegen, Netherlands; h Section of Pediatric Infectious Diseases, Laboratory of Medical Immunology, Radboud Institute of Molecular Life Sciences, Radboud University Medical Center, Nijmegen, Netherlands; i Department of Infectious Diseases, Leiden University Medical Center, Leiden, Netherlands; j Department of Medicine and Radboud Center for Infectious Diseases, Radboud University Medical Center, Nijmegen, Netherlands; k Department for Genomics & Immunoregulation, Life and Medical Sciences Institute, University of Bonn, Bonn, Germany; NWZ Alkmaar; Radboud; Radboud; Radboud; Radboud; Radboud; JBZ Den Bosch; UMC Utrecht; Leiden UMC; Leiden UMC; UMC Utrecht; Radboud; UMC Utrecht; HZ Den Haag; Radboud; Leiden UMC; NWZ Alkmaar; Erasmus MC; Leiden UMC; Radboud; UMC Utrecht; Radboud; CWZ Nijmegen; UMC Utrecht; Fondazione Biotecnopolo di Siena

**Keywords:** SARS-CoV-2, COVID-19, Bacillus Calmette-Guerin vaccine, randomized placebo-controlled clinical trial, health care workers

## Abstract

Bacillus Calmette-Guerin (BCG) vaccination has been hypothesized to reduce severe acute respiratory syndrome coronavirus 2 (SARS-CoV-2) infection, severity, and/or duration via trained immunity induction. Health care workers (HCWs) in nine Dutch hospitals were randomized to BCG or placebo vaccination (1:1) in March and April 2020 and followed for 1 year. They reported daily symptoms, SARS-CoV-2 test results, and health care-seeking behavior via a smartphone application, and they donated blood for SARS-CoV-2 serology at two time points. A total of 1,511 HCWs were randomized and 1,309 analyzed (665 BCG and 644 placebo). Of the 298 infections detected during the trial, 74 were detected by serology only. The SARS-CoV-2 incidence rates were 0.25 and 0.26 per person-year in the BCG and placebo groups, respectively (incidence rate ratio, 0.95; 95% confidence interval, 0.76 to 1.21; *P* = 0.732). Only three participants required hospitalization for SARS-CoV-2. The proportions of participants with asymptomatic, mild, or moderate infections and the mean infection durations did not differ between randomization groups. In addition, unadjusted and adjusted logistic regression and Cox proportional hazards models showed no differences between BCG and placebo vaccination for any of these outcomes. The percentage of participants with seroconversion (7.8% versus 2.8%; *P* = 0.006) and mean SARS-CoV-2 anti-S1 antibody concentration (13.1 versus 4.3 IU/mL; *P* = 0.023) were higher in the BCG than placebo group at 3 months but not at 6 or 12 months postvaccination. BCG vaccination of HCWs did not reduce SARS-CoV-2 infections nor infection duration or severity (ranging from asymptomatic to moderate). In the first 3 months after vaccination, BCG vaccination may enhance SARS-CoV-2 antibody production during SARS-CoV-2 infection.

## INTRODUCTION

The Bacillus Calmette-Guerin (BCG) vaccine is a widely used live-attenuated vaccine against tuberculosis. Routine childhood and/or health care worker (HCW) vaccination is performed worldwide, except in some countries with a low tuberculosis burden, including the Netherlands. New interest in BCG has arisen after recent studies showed that BCG vaccination also has nonspecific protective effects against other respiratory tract infections due to epigenetic and metabolic reprogramming of innate immune cells (1, 2). This process is termed “trained immunity.” It was first observed in children in high-infection prevalence settings (3), but later also in observational studies in adults (4, 5) and in adults challenged with malaria or live-attenuated yellow fever or influenza vaccination after BCG vaccination (6–8).

In the first year of the severe acute respiratory syndrome coronavirus 2 (SARS-CoV-2) pandemic, testing and contact-tracing capacity, as well as personal protection equipment supplies, were limited and SARS-CoV-2 vaccines were not yet available. These conditions raised challenges, especially for HCWs and vulnerable adults. HCWs were burdened with a high risk of exposure and infection while the demand for medical personnel increased (9–11), and vulnerable adults were at high risk of hospitalization and death. However, BCG vaccines were available. In this context, several randomized placebo-controlled BCG trials with 2019 coronavirus disease (COVID-19) endpoints were initiated during the pandemic to determine whether BCG vaccination reduced SARS-CoV-2 infection and/or infection severity or duration.

We recently published the results of the BCG-Corona trial in 1,511 Dutch HCWs (12). The primary endpoint of this trial was absenteeism for any reason. Secondary endpoints were, among others, participant-reported positive SARS-CoV-2 tests and symptomatic respiratory infections. None of these endpoints differed statistically significantly between the BCG and placebo groups. However, while participants were instructed to always get tested in case of COVID-19-like symptoms, they may not always have done so, especially in the case of mild infections. Furthermore, asymptomatic infections likely went unnoticed. We therefore also collected blood samples for SARS-CoV-2 antibody testing at 3 to 6 months and 12 months postvaccination. This uncovered 74 additional infections in addition to the 224 participant-reported infections. Our aim was to determine whether BCG vaccination compared to placebo vaccination reduced SARS-CoV-2 infection acquisition, severity, and/or duration using this most comprehensive data set in the field to date, with endpoints detected by both self-reporting and serology and with daily data on symptoms.

## RESULTS

### Participant flow and baseline characteristics.

**FIG 1:**
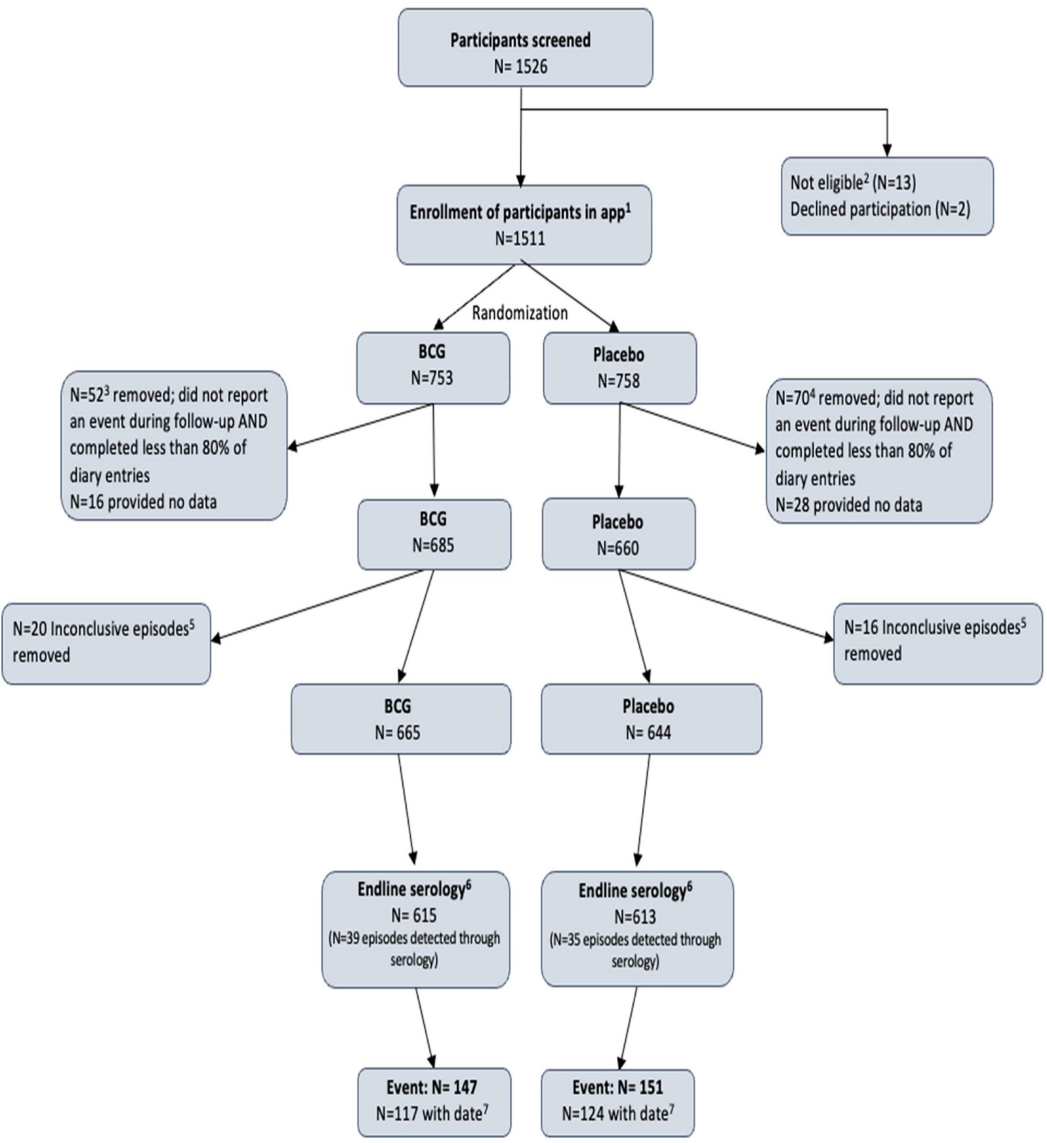
BCG-Corona Study flow. Footnotes: (1) The first participant was enrolled on 24 March 2020 and the last participant on 23 April 2020. The last day of follow-up in the app was 27 March 2021, with additional participant information obtained via an online questionnaire to cover the period between 28 March 2021 and the last blood sampling date, if applicable. (2) Thirteen participants were not eligible: pregnancy (*N* = 4), severely immunocompromised state (*N* = 1), malignancy or lymphoma in the last 2 years (*N* = 1), active viral or bacterial infection (*N* = 2), recent or expected vaccination (*N*= 3), not hospital personnel caring for COVID-19 patient (*N* = 1), or Mycobacterium tuberculosis infection history (*N* = 1). (3) Of the 52 participants in the BCG group removed due to less than 80% diary app completion and no evidence of infection, 19 terminated study participation early at their own request, 10 became complete nonresponders prior to the end of the study for unknown reasons, 2 went on pregnancy leave, and 21 entered data inconsistently. An additional 16 participants never entered any data in the app. (4) Of the 70 participants in the placebo group removed due to less than 80% diary app completion and no evidence of infection, 33 terminated study participation early at their own request, 10 became complete nonresponders prior to the end of the study for unknown reasons, 6 terminated hospital employment, 3 went on pregnancy leave, and 18 entered data inconsistently. An additional 28 participants never entered any data in the app. The percentage of participants that completed less than 80% of the diary entries in the BCG group was not statistically different from the percentage in the placebo group (chi-squared *P* = 0.283). (5) An inconclusive episode was defined as anti-N seropositivity but not anti-S1 seropositivity at the end of period 2. The precentage of participants that we removed from the BCG group because they had inconclusive episodes only was not statistically different from the percentage in the placebo group (chi-squared *P* = 0.599). (6) *N* is the number of participants that participated in at least one sampling round at the end of the follow-up (chi-squared *p* = 0.237). (7) Events with a date were included in all analyses and events without a date in logistic regressions only.

Between 24 March and 23 April 2020, 1,526 HCWs were screened and 1,511 were randomized, with 753 HCWs in the BCG group and 758 in the placebo group (Fig. 1). Participants with less than 80% app completion and no evidence of infection (see Fig. S1 in the supplemental material), or with inconclusive episodes only, were removed (68 and 20 in the BCG group and 98 and 16 in the placebo group, respectively). The analysis population therefore consisted of 1,309 participants: 665 in the BCG group and 644 in the placebo group. Of the analysis population, 82.9% in the BCG group and 85.7% in the placebo group provided a blood sample in the second sampling round. An additional 9.6% and 9.5%, respectively, provided a blood sample in the first but not in the second sampling round. None of the data availability characteristics differed significantly between the randomization groups (Fig. 1).

10.1128/mbio.00356-23.1 FIG S1 Completeness of smartphone diary application entries by all randomized participants (*N* = 1,511) (A) and by all participants that experienced an event (*N* = 298) (B). Download FIG S1, DOCX file, 0.1 MB. Copyright © 2023 Claus et al. 2023 Claus et al. https://creativecommons.org/licenses/by/4.0/ This content is distributed under the terms of the Creative Commons Attribution 4.0 International license.

**FIG 2:**
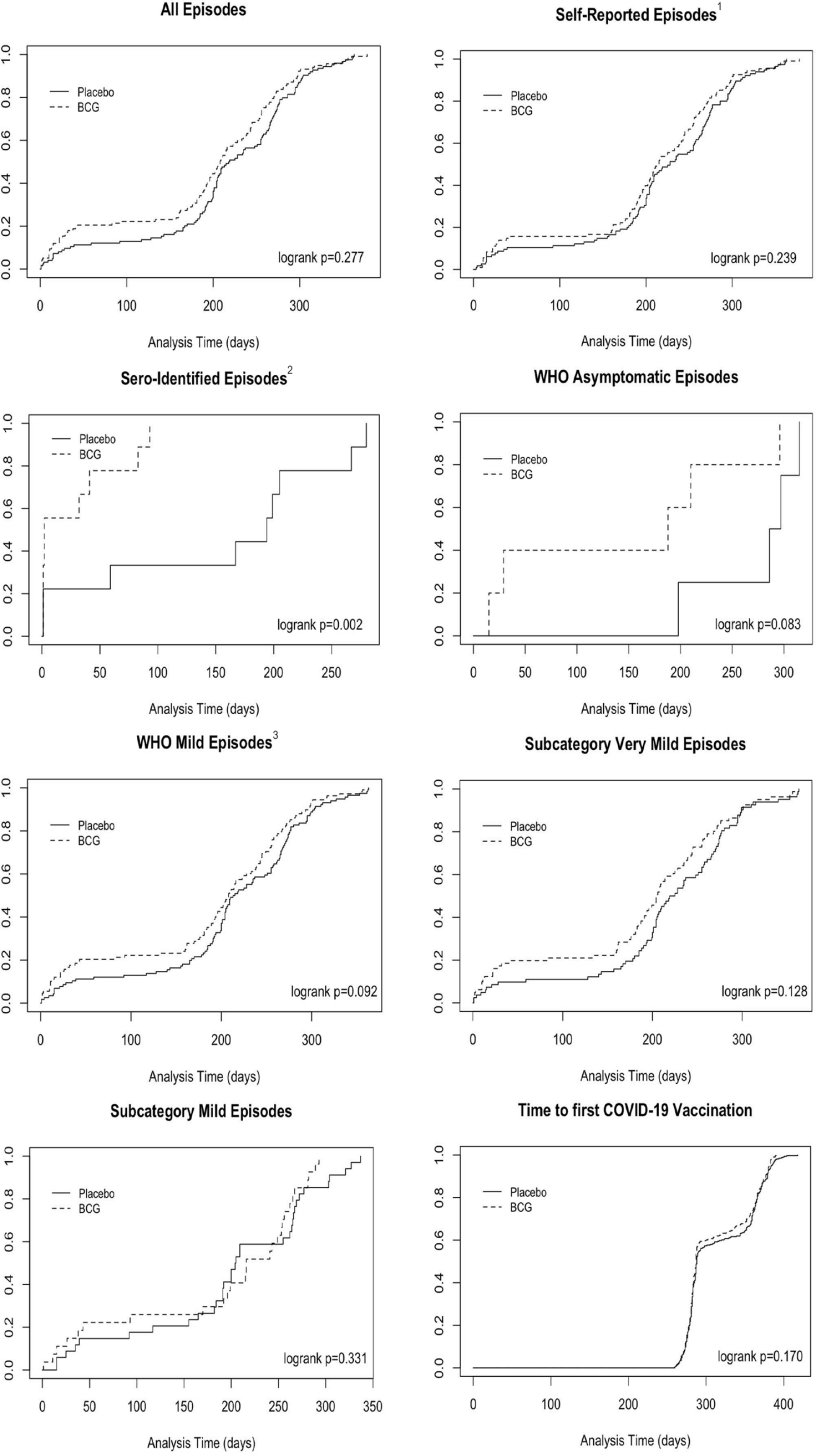
Time-to-first event curves. Footnotes: (1) Self-reported episodes included proven episodes with and without seroconversion. (2) Sero-identified episodes included all possible episodes detected through serology that could be linked to a symptomatic period and therefore had a known episode infection date. (3) WHO mild episodes were further subcategorized into very mild and mild episodes (definitions can be found in Text S1 in the supplemental material). The WHO moderate episodes are not shown in time-to-event curves because there were only three episodes.

The majority of the 1,309 participants (74.4%) were female, with no difference between groups (Table 1). The age of participants ranged between 18 and 67 years, with mean ages of 41.8 years in the BCG group and 43.2 years in the placebo group. Job-related characteristics were also similar between groups: 49.3% of the participants were nurses and 53.1% reported to have direct patient contact at least 75% of their work hours. Household size, smoking behavior, histories of BCG or recent influenza or other vaccinations, ever having had a positive tuberculosis test, having had a respiratory tract infection last winter, and chronic comorbidities were well-balanced between groups. The baseline characteristics of the analysis population were comparable to those of the entire randomized population (Table S1A). Some significant differences in baseline characteristics were observed between participants recruited at the three core hospitals versus the six other hospitals, but this did not affect the BCG-placebo comparisons because randomization was stratified by hospital (Table S1B). About half of the participants (47.2% in the BCG group and 50.6% in the placebo group; *P* = 0.218) received at least one dose of one of five different COVID-19 vaccines between 6 January 2021 and the end of follow-up. The time to first COVID-19 vaccination did not differ between the randomization groups (Fig. 2).

**TABLE 1:** Baseline characteristics of the analysis population by study group

Characteristic	No. (%) with characteristic in:			*P* ^*a*^
	BCG group (*N* = 665)	Placebo group (*N* = 644)	Total (*N* = 1,309)	
Recruitment site				0.997
Radboud University Medical Center Nijmegen	186 (28.0)	177 (27.5)	363 (27.7)	
University Medical Center Utrecht	167 (25.1)	159 (24.7)	326 (24.9)	
Noordwest Hospital Alkmaar	148 (22.3)	141 (21.9)	289 (22.1)	
Haga Hospital Den Haag	42 (6.3)	40 (6.2)	82 (6.3)	
Canisius-Wilhelmina Hospital Nijmegen	31 (4.7)	32 (5.0)	63 (4.8)	
Sint Maartenskliniek Nijmegen	24 (3.6)	30 (4.7)	54 (4.1)	
Leiden University Medical Center	25 (3.8)	24 (3.7)	49 (3.7)	
Jeroen Bosch Hospital Den Bosch	23 (3.5)	21 (3.3)	44 (3.4)	
Erasmus Medical Center Rotterdam	19 (2.9)	20 (3.1)	39 (3.0)	
Mean (SD) age (yrs)^*b*^	41.79 (12.66)	43.21 (12.73)	42.49 (12.71)	**0.043**
Female sex	501 (75.4)	473 (73.4)	974 (74.4)	0.471
Mean (SD) no. of additional household members	1.96 (1.38)	1.86 (1.40)	1.91 (1.39)	0.184
Smoking status				0.218
Current	50 (7.5)	45 (7.0)	95 (7.3)	
Former	213 (32.0)	180 (28.0)	393 (30.0)	
Never	402 (60.5)	419 (65.1)	821 (62.7)	
Hospital department				0.740
Urgent care	36 (5.4)	39 (6.1)	75 (5.7)	
Internal medicine^*c*^	110 (16.6)	93 (14.4)	203 (15.5)	
Intensive or medium care	64 (9.6)	63 (9.8)	127 (9.7)	
Other	455 (68.4)	449 (69.7)	904 (69.1)	
Job function				0.507
Doctor	145 (21.8)	149 (23.1)	294 (22.5)	
Nurse	329 (49.5)	316 (49.1)	645 (49.3)	
Paramedic	112 (16.8)	92 (14.3)	204 (15.6)	
Support personnel	79 (11.9)	87 (13.5)	166 (12.7)	
Scheduled to work on COVID ward				0.878
No	198 (29.8)	188 (29.2)	386 (29.5)	
Yes	425 (63.9)	411 (63.8)	836 (63.9)	
Unknown	42 (6.3)	45 (7.0)	87 (6.7)	
% work hours with patient contact				0.212
0–25	95 (14.3)	112 (17.4)	207 (15.8)	
26–50	107 (16.1)	83 (12.9)	190 (14.5)	
51–75	113 (17.0)	104 (16.1)	217 (16.6)	
75+	350 (52.6)	345 (53.6)	695 (53.1)	
History of BCG vaccination	118 (17.8)	108 (16.8)	226 (17.3)	0.694
Past tuberculosis test results^*d*^				0.055
Tested negative	447 (67.2)	442 (68.6)	889 (67.9)	
Tested positive (either or both)	55 (8.3)	67 (10.4)	122 (9.3)	
Never tested	153 (23.0)	133 (20.6)	286 (21.8)	
Unknown (both)	10 (1.5)	2 (0.3)	12 (0.9)	
Respiratory infection in winter 2019–2020				0.525
No	483 (72.6)	461 (71.6)	944 (72.1)	
Yes, with fever	61 (9.2)	52 (8.1)	113 (8.6)	
Yes, no fever	121 (18.2)	131 (20.3)	252 (19.3)	
Influenza vaccination in winter 2020–2021				0.272
Yes	328 (49.3)	346 (53.7)	674 (51.5)	
No	215 (32.4)	193 (30.0)	408 (31.2)	
Missing	122 (18.3)	105 (16.3)	227 (17.3)	
Any other vaccination in past yr^*e*^	73 (11.0)	63 (9.8)	136 (10.4)	0.537
Current use of antihypertensive medication	46 (6.9)	44 (6.8)	90 (6.9)	1
History of cardiovascular disease	13 (2.0)	17 (2.6)	30 (2.3)	0.520
Current use of antidiabetes medication	3 (0.5)	4 (0.6)	7 (0.5)	0.966
History of asthma	48 (7.2)	43 (6.7)	91 (7.0)	0.782
History of hay fever	207 (31.2)	174 (27.0)	381 (29.1)	0.115
History of other pulmonary disease	13 (2.0)	16 (2.5)	29 (2.2)	0.643
Any lung disease (previous three combined)	229 (34.5)	202 (31.4)	431 (32.9)	0.262
Positive SARS-CoV-2 test prior to baseline	1 (0.2)	0 (0.16)	1 (0.1)	1
At least 1 dose COVID-19 vaccine during follow-up^*f*^	313 (47.2)	326 (50.6)	639 (48.8)	0.218

a Chi-squared tests for categorical variables and Wilcoxon rank sum test for continuous variables.

b The 1.42 years difference in mean age was statistically significant, but we believe that it was not relevant in this context.

c Internal medicine includes the pulmonology and infectious disease departments.

d Tuberculosis tests included the Mantoux and/or TB QuantiFERON tests.

e The following other vaccinations were reported: diphtheria-tetanus-pertussis-polio (DTaP-IPV), hepatitis A, hepatitis B, yellow fever, typhoid, rabies, mumps-measles-rubella, meningococcal, pneumococcal, Haemophilus influenzae type B, Ebola, tick-borne encephalitis, human papillomavirus, and unknown.

f In the Netherlands, the SARS-CoV-2 vaccines available during the study period were Spikevax (Moderna Biotech, Cambridge, MA, USA), Comirnaty (Pfizer/BioNTech, New York, NY, USA), Vaxzevria (AstraZeneca AB, Sodertalje, Sweden), and Jcovden (Janssen Vaccines, Leiden, Netherlands). In addition, one participant received an experimental vaccine by CureVac N.V. in a clinical trial setting. This vaccine was never marketed due to insufficient efficacy. See Table S1 in the supplemental material for the number and percentage of participations in each vaccine category by randomization group.

10.1128/mbio.00356-23.4 TABLE S1 Baseline characteristics of the randomized population (A) and of core hospitals versus other hospitals (B). Download Table S1, DOCX file, 0.05 MB. Copyright © 2023 Claus et al. 2023 Claus et al. https://creativecommons.org/licenses/by/4.0/ This content is distributed under the terms of the Creative Commons Attribution 4.0 International license.

### Descriptive outcomes by randomization group.

SARS-CoV-2 infections over calendar time in the study population mirrored epidemic waves in the Netherlands (Fig. S2). Only one participant (in the BCG group) reported to have had a positive SARS-CoV-2 test prior to randomization. A total of 298 infections (not including 4 second infections) were detected during the trial, of which 224 were participant reported and 74 detected by serology only (Table 2). Overall, 147/665 participants (22.1%) in the BCG group and 151/644 participants (23.4%) in the placebo group experienced an infection during follow-up (*P* = 0.608). The SARS-CoV-2 incidence was 0.25 per person-year in the BCG group and 0.26 per person-year in the placebo group (incidence rate ratio, 0.95; 95% confidence interval [CI], 0.76 to 1.21; *P* = 0.732) (Table S2).

**TABLE 2:** Outcomes during follow-up, by randomization group

Outcome	BCG (*N* = 665)	Placebo (*N =* 644)	Total (*N =* 1,309)	*P* ^*a*^
Reported a positive test during follow-up, *n* (%)	108 (16.3)	116 (18.0)	224 (17.1)	0.528
All episodes based on reported tests and serology during follow-up, *n* (%)	147 (22.1)	151 (23.4)	298 (22.8)	0.608
Episode severity (WHO definitions), *n* (%)				0.806
Asymptomatic	23 (15.6)	19 (12.6)	42 (14.1)	
Mild	108 (73.5)	116 (76.8)	224 (75.2)	
Moderate	1 (0.7)	2 (1.3)	3 (1.0)	
Unknown	15 (10.2)	14 (9.3)	29 (9.7)	
Episode severity with subcategories, *n* (%)^*b*^				0.823
Asymptomatic	23 (15.6)	19 (12.6)	42 (14.1)	
Very mild	81 (55.1)	82 (54.3)	163 (54.7)	
Mild	27 (18.4)	34 (22.5)	61 (20.5)	
Moderate	1 (0.7)	2 (1.3)	3 (1.0)	
Unknown	15 (10.2)	14 (9.3)	29 (9.7)	
Long-term loss of smell and/or taste, *n* (%)^*c*^	4 (2.8)	8 (5.4)	12 (4.1)	0.431
Developed long-COVID, *n* (%)^*d*^	15 (10.8)	12 (8.3)	27 (9.5)	0.616
Mean (SD) duration of acute episode^*e*^	12.24 (10.93)	13.38 (11.96)	12.84 (11.47)	0.590
Mean (SD) duration of acute episode, excluding asymptomatic and long-COVID episodes^*f*^	15.44 (10.06)	15.90 (11.40)	15.68 (10.76)	0.890
Episode type, *n* (%)				**0.008**
Participant-reported with seroconversion	71 (48.3)	78 (51.7)	149 (50.0)	
Participant-reported with no seroconversion	12 (8.2)	27 (17.9)	39 (13.1)	
Participant-reported and no serology	25 (17.0)	11 (7.3)	36 (12.1)	
Identified by seroconversion only	39 (26.5)	35 (23.2)	74 (24.8)	
Period that episode occurred in and data source, *n* (%)^*g*^				0.075, 0.108
Period 1: participant reported	28 (49.1)	24 (70.6)	52 (57.1)	
Period 1: sero-identified only	29 (50.9)	10 (29.4)	39 (42.9)	
Period 2: participant reported	80 (90.0)	92 (80.7)	172 (84.7)	
Period 2: sero-identified only	9 (10.0)	22 (19.3)	31 (15.3)	
Unknown period	1 (0.7)	3 (2.0)	4 (1.3)	
Antibody results for participants with samples available^*h*^				
No. (%) with anti-S1 antibodies at mo. 3; total group *N*	27 (7.8); 344	9 (2.8); 325	36 (5.4); 669	**0.006**
Mean (SD) anti-S1 concn at mo. 3 (IU/mL)	13.05 (64.87)	4.34 (23.61)	8.82 (49.50)	**0.023**
No.^*i*^ (%) with anti-S1 antibodies at mo. 6; total group *N*	21 (8.8); 238	16 (7.0); 229	37 (7.9); 467	0.573
Mean (SD) anti-S1 concn at mo. 6 (IU/mL)	11.37 (50.74)	13.67 (79.57)	12.50 (66.40)	0.709
No.^*j*^ (%) with anti-S1 antibodies at round 1; total group *N*	48 (8.2); 582	25 (4.5); 553	73 (6.4); 1,135	**0.015**
Mean (SD) anti-S1 concn at Round 1 (IU/mL)	12.37 (59.45)	8.21 (54.44)	10.34 (57.08)	0.220
No.^*k*^ (%) with anti-S1 + anti-N antibodies at round 2; total group *N*	75 (13.6); 551	86 (15.6); 552	161 (14.6); 1,103	0.401
Mean (SD) anti-S1 concn at round 2 (IU/mL)	902.51 (2,690.39)	1,007.96 (2,765.13)	955.28 (2,727.32)	0.521
Mean (SD) anti-N concn at round 2 (IU/mL)	17.61 (72.10)	16.28 (68.56)	16.94 (70.32)	0.752

a Based on chi-square test for categorical variables and Wilcoxon rank sum test for means. *P* values of <0.05 are indicated in boldface.

b We further subdivided the WHO “mild” category into very mild and mild subcategories (see the definitions in Text S1 in the supplemental material).

c Reported stand-alone loss of smell and/or taste for at least 60 days after the end of acute infection episode. The denominators are 141, 149, and 290 for BCG, placebo, and total, respectively, because 6, 2, and 8 episodes that had not yet reached 60 days of stand-alone loss of smell and/or taste but were ongoing at the end of follow-up were excluded.

d Reported symptoms other than stand-alone loss of smell and/or taste for at least 60 days after the end of acute infection episode. The denominators are 139, 144, and 283 for BCG, placebo, and total, respectively, because 8, 7, and 15 episodes that had not yet reached 60 days of lingering symptoms other than stand-alone loss of smell and/or taste but were ongoing at the end of follow-up were excluded.

e *N* = 231 episodes. The following episodes were removed from the calculations: 27 long-COVID cases, 15 episodes that had not yet reached the long-COVID definition but were ongoing at the end of follow-up, and 25 episodes that could not be matched with one specific symptoms episode and therefore had an unknown episode duration. In cases of lingering stand-alone loss of smell and/or taste, only the duration of the acute infection episode (with respiratory tract symptoms) was included.

f *N* = 189 episodes. The same episodes as listed under footnote e were removed from the calculations, as were 42 asymptomatic episodes with a duration of 0 days.

g Period 2 data included period 1 if only one sample (at the end of period 2) was taken. Four episodes (one in the BCG group and three in the placebo group) could not be assigned to a period. The first *P* value compares the distribution between participant-reported and sero-identified episodes in the BCG and placebo groups during period 1, and the second *P* value for episodes in period 2. Four episodes (one in the BCG group and three in the placebo group) could not be assigned to a period and were excluded from both statistical tests.

h *N* is the total participants in the denominator. Participants who did not volunteer a blood sample at the given sampling round or were not offered blood sampling in their hospital were removed. Comparison of baseline characteristics between serology cohorts at month 3 (core hospitals) and month 6 (other hospitals) are shown in Table S1B in the supplemental material.

i Includes core hospital participants who had missed their month 3 venipuncture visit and agreed to provide a fingerprick sample at home instead.

j One participant volunteered a blood sample at both months 3 and 6; however, they were only included once for the round 1 results.

k Includes participants who had received at least one dose of a COVID-19 vaccine. They were equally distributed among the randomization groups (Table 1; Fig. 2).

10.1128/mbio.00356-23.2 FIG S2 SARS-CoV-2 infections over calendar time in the study population for all infections (A), by infection severity (WHO definitions with subcategories) (B), by recruitment site (C), and in the Netherlands as a whole (D). Download FIG S2, DOCX file, 0.5 MB. Copyright © 2023 Claus et al. 2023 Claus et al. https://creativecommons.org/licenses/by/4.0/ This content is distributed under the terms of the Creative Commons Attribution 4.0 International license.

10.1128/mbio.00356-23.5 TABLE S2 Additional outcomes during follow-up in the analysis population. Download Table S2, DOCX file, 0.04 MB. Copyright © 2023 Claus et al. 2023 Claus et al. https://creativecommons.org/licenses/by/4.0/ This content is distributed under the terms of the Creative Commons Attribution 4.0 International license.

In the BCG and placebo groups, respectively, 0.7% and 1.3% of first infections were moderate, 73.5% and 76.8% were mild (of which 18.4% and 22.5% were mild and 55.1% and 54.3% were very mild), and 15.6% and 12.6% were asymptomatic (no statistically significant differences) (Table 2). As expected, most symptomatic infections were identified via participant reporting: 92.0% of mild and 100% of moderate episodes. In contrast, 76.2% of the asymptomatic episodes were identified by serology only. Fifteen episodes in the BCG group (10.8%) and 12 in the placebo group (8.3%) reached the long-COVID definition during follow-up (*P* = 0.616). In addition, four (2.8%) and eight (5.4%) episodes in the BCG and placebo groups, respectively, resulted in long-term loss of smell and/or taste (*P* = 0.431) (Table 2). The mean episode durations of acute episodes (not including long-COVID or asymptomatic episodes) were 15.4 days (standard deviation [SD], 10.1 days) in the BCG group and 15.9 days (SD 11.4 days) in the placebo group (*P* = 0.890) (Table 2, Fig. S3). None of the different types of symptoms, nor their individual severities, differed between randomization groups (Table S2).

10.1128/mbio.00356-23.3 FIG S3 Infection episode duration distributions by randomization group. Download FIG S3, DOCX file, 0.1 MB. Copyright © 2023 Claus et al. 2023 Claus et al. https://creativecommons.org/licenses/by/4.0/ This content is distributed under the terms of the Creative Commons Attribution 4.0 International license.

About a quarter of all infections (24.8%) was identified by serology only, and this percentage was non-significantly higher in the BCG group in period 1 (50.9%) compared to the BCG group in period 2 (10.0%) and or to the placebo group in both periods (29.4% and 19.3%) (Table 2). Seroidentified episodes accumulated faster in the BCG than in the placebo group (logrank *P* = 0.002, Fig. 2). The percentage of participants with seroconversion was higher in the BCG group than in the placebo group at month 3 (7.8% versus 2.8%; *P* = 0.006), but not at month 6 (8.8% versus 7.0%; *P* = 0.573) or month 12 (13.6% versus 15.6%; *P* = 0.401). Similarly, the mean anti-S1 antibody concentration was higher in the BCG group than in the placebo group at month 3, but there were no differences in anti-S1 or anti-N antibody concentrations between the groups at months 6 or 12 (Table 2).

### Logistic regression and Cox proportional hazards models.

Unadjusted and adjusted logistic regression models showed no differences between BCG and placebo vaccination in cumulative incidence of any SARS-CoV-2 infection or of asymptomatic, mild (subdivided into mild and very mild), or moderate infections separately (Table 3). No differences were seen in Cox proportional hazards time-to-first-event models with these same outcomes, either (Table 3, Fig. 2). Younger age, various job-related characteristics, past BCG vaccination, and current use of hypertension medication were associated with increased odds or hazards of acquiring infection (Table S3) and were retained in all multivariable models (Table 3, Table S4). A larger household size was associated with increased odds or hazards in some but not all models. In terms of job-related characteristics, being a nurse or support staff (compared to a doctor), having a larger percentage of work hours with patient contact, and expectation to work on a COVID ward were associated with higher odds or hazards of infection or infection severity, and working in the intensive or medium care departments (compared to the urgent care department) were associated with lower odds or hazards. Recruitment site, enrollment week, sex, ever having tested positive for tuberculosis, current use of antidiabetic medication, history of pulmonary disease, and history of cardiovascular disease were not associated with an outcome in any of the models. All sensitivity analyses showed similar results (Tables S5 and S6).

**TABLE 3:** Logistic regression and Cox proportional hazards models

Statistical models^*a*^	Covariates	OR (95% CI)	*P* value
Logistic regression			
Univariate, all episodes	BCG vs placebo	0.93 (0.72–1.20)	0.563
Univariate, WHO asymptomatic only	BCG vs placebo	1.15 (0.62–2.16)	0.654
Univariate, WHO mild only	BCG vs placebo	0.89 (0.66–1.18)	0.413
Univariate, subcategory very mild only	BCG vs placebo	0.94 (0.67–1.31)	0.715
Univariate, subcategory mild only	BCG vs placebo	0.76 (0.45–1.27)	0.291
Univariate, WHO moderate only	BCG vs placebo	0.48 (0.02–4.98)	0.545
Multivariable model, all episodes^*b*^	BCG vs placebo	0.85 (0.65–1.12)	0.249
	Age (yrs)	0.98 (0.97–0.99)	**0.001**
	Household size (per additional member)	1.10 (1.00–1.21)	**0.040**
	Function		
	Doctor	—	—
	Nurse	1.84 (1.26–2.72)	**0.002**
	Paramedic	1.47 (0.90–2.40)	0.120
	Support staff	2.11 (1.19–3.71)	**0.010**
	% of work hours with patient contact		
	0–25	—	—
	26–50	1.80 (0.99–3.31)	0.077
	51–75	1.71 (0.95–3.14)	0.056
	75+	2.47 (1.46–4.31)	**0.001**
	Hospital department		
	Urgent Care	—	—
	Internal Medicine	1.58 (0.87–2.93)	0.139
	Intensive or Medium Care	0.64 (0.32–1.28)	0.201
	Other	0.94 (0.55–1.66)	0.822
	COVID ward		
	No	—	—
	Yes	1.58 (1.12–2.27)	**0.011**
	Unknown	1.48 (0.80–2.68)	0.199
	Past BCG vaccination	1.70 (1.15–2.48)	**0.007**
	Current hypertension medication use	1.94 (1.15–3.21)	**0.011**
Multinomial logistic regression			
Univariate, all episodes (WHO severity definitions^*c*^)	BCG vs placebo		
	No infection	—	—
	Asymptomatic	1.15 (0.62–2.14)	0.655
	Mild	0.89 (0.66–1.18)	0.413
	Moderate	0.48 (0.04–5.27)	0.545
Multivariable model, all episodes (WHO severity definitions^*b*^^,^^*c*^^,^^*d*^)	BCG vs placebo		
	No infection	—	—
	Asymptomatic	1.12 (0.60–2.09)	0.729
	Mild	0.82 (0.61–1.10)	0.183
	Moderate	0.41 (0.03–5.85)	0.509
Univariate, all episodes, with subcategories^*e*^	BCG vs placebo		
	No infection	—	—
	Asymptomatic	1.15 (0.62–2.14)	0.655
	Very mild	0.94 (0.68–1.31)	0.715
	Mild	0.76 (0.45–1.27)	0.292
	Moderate	0.48 (0.04–5.28)	0.546
Multivariable model, all episodes, with subcategories^*b*^^,^^*e*^^,^^*f*^	BCG vs placebo		
	No infection	—	—
	Asymptomatic	1.13 (0.60–2.11)	0.711
	Very mild	0.87 (0.62–1.22)	0.416
	Mild	0.72 (0.43–1.21)	0.230
	Moderate	0.64 (0.05–8.24)	0.729
Cox proportional hazards^*g*^^,^^*h*^			
Univariate, all episodes	BCG vs placebo	0.92 (0.72–1.19)	0.521
Univariate, WHO asymptomatic only	BCG vs placebo	1.19 (0.32–4.44)	0.793
Univariate, WHO mild only	BCG vs placebo	0.91 (0.70–1.18)	0.468
Univariate, subcategory very mild only	BCG vs placebo	0.96 (0.70–1.30)	0.769
Univariate, subcategory mild only	BCG vs placebo	0.76 (0.46–1.26)	0.293
Univariate, WHO moderate only	BCG vs placebo	0.48 (0.04–5.25)	0.544
Multivariable Cox proportional hazards model, all episodes^*b*^	BCG vs placebo	0.86 (0.66–1.11)	0.238
	Age (yrs)	0.98 (0.97–0.99)	**0.002**
	Household size (per additional member)	1.10 (1.01–1.19)	**0.024**
	Function		
	Doctor	—	—
	Nurse	1.73 (1.19–2.52)	**0.004**
	Paramedic	1.37 (0.85–2.21)	0.195
	Support staff	2.05 (1.19–3.53)	**0.010**
	% of work hours with patient contact		
	0–25	—	—
	26–50	1.85 (1.02–3.35)	**0.044**
	51–75	1.81 (1.00–3.27)	0.050
	75+	2.30 (1.33–3.95)	**0.003**
	Hospital department		
	Urgent Care	—	—
	Internal Medicine	1.41 (0.81–2.45)	0.222
	Intensive or Medium Care	0.47 (0.23–0.97)	**0.040**
	Other	0.92 (0.55–1.54)	0.753
	COVID ward		
	No	—	—
	Yes	1.50 (1.06–2.12)	**0.021**
	Unknown	1.42 (0.80–2.52)	0.230
	Past BCG vaccination	1.58 (1.11–2.25)	**0.011**
	Current hypertension medication use	1.90 (1.22–2.98)	**0.005**

a *N* = 1,309 with cumulative SARS-CoV-2 infections as the endpoint.

b Modeling is described in Materials and Methods and the supplemental material. In addition to the variables listed in this table, the following variables were considered for inclusion in each model: recruitment site, enrollment week, sex, smoking status, ever having tested positive for tuberculosis, current use of antidiabetes medication, and history of pulmonary disease or cardiovascular disease.

c *N* = 1,309 with cumulative WHO-defined asymptomatic, mild, or moderate SARS-CoV-2 infections as endpoints.

d Covariates retained in the model included age in years, hospital department, percentage of work hours with patient contact, past BCG vaccination, and current use of hypertension medication.

e *N* = 1,309 with cumulative asymptomatic, very mild, mild, or moderate SARS-CoV-2 infections as endpoints.

f Covariates retained in model included age in years, hospital function and department, expected to work in COVID ward, past BCG vaccination, and current use of hypertension medication.

g *N* = 1,252 participants and *N* = 241 events with a known date; *N* = 57 events that were excluded in all survival analyses due to a lack of event date (all asymptomatic infections that were identified by serology only or insufficient data).

h Cumulative SARS-CoV-2 infection as the endpoint.

10.1128/mbio.00356-23.6 TABLE S3 Univariable regression models with SARS-CoV-2 infection as outcome. Download Table S3, DOCX file, 0.04 MB. Copyright © 2023 Claus et al. 2023 Claus et al. https://creativecommons.org/licenses/by/4.0/ This content is distributed under the terms of the Creative Commons Attribution 4.0 International license.

10.1128/mbio.00356-23.7 TABLE S4 Multinomial logistic regression model with infection severity as outcome. Download Table S4, DOCX file, 0.04 MB. Copyright © 2023 Claus et al. 2023 Claus et al. https://creativecommons.org/licenses/by/4.0/ This content is distributed under the terms of the Creative Commons Attribution 4.0 International license.

10.1128/mbio.00356-23.8 TABLE S5 Sensitivity analysis, with participants with less than 80% diary app completion and no evidence of infection added back into sample, assuming that they never had an infection. Download Table S5, DOCX file, 0.04 MB. Copyright © 2023 Claus et al. 2023 Claus et al. https://creativecommons.org/licenses/by/4.0/ This content is distributed under the terms of the Creative Commons Attribution 4.0 International license.

10.1128/mbio.00356-23.9 TABLE S6 Sensitivity analysis, assuming inconclusive infections were or were not true infections. Download Table S6, DOCX file, 0.04 MB. Copyright © 2023 Claus et al. 2023 Claus et al. https://creativecommons.org/licenses/by/4.0/ This content is distributed under the terms of the Creative Commons Attribution 4.0 International license.

## DISCUSSION

This is the most comprehensive analysis of a placebo-controlled BCG trial with SARS-CoV-2 endpoints to date, including both participant-reported positive tests as well as serology to identify infections and in-depth characterization of infection episodes using daily symptoms data. Serology detected an additional 74 endpoints on top of the 224 participant-reported endpoints. Unadjusted and adjusted logistic regression (cumulative incidence) and Cox proportional hazards (time-to-first-event) models showed no differences between BCG and placebo vaccination for SARS-CoV-2 infections of any severity, nor for asymptomatic, mild (subdivided into mild and very mild), or moderate infections separately. Mean infection durations and the proportions of participants with infections who developed long-COVID or long-term loss of smell and/or taste did not differ between the randomization groups either.

Our findings corroborated the results of several placebo-controlled BCG trials with SARS-CoV-2 endpoints. A South African trial in HCWs (*N* = 1,000) (13) and two Dutch trials in community-dwelling elderly populations (*N* = 2,014) (14) and vulnerable elderly populations (*N* = 6,112) (15) found no differences between BCG and placebo vaccination in participant-reported positive SARS-CoV-2 tests, COVID-19 hospitalizations or deaths, or any symptomatic respiratory tract infection in the year following vaccination. The South African trial did not detect a difference in the cumulative incidence of SARS-CoV-2 seroconversion either, but it is unclear how seroconversion was defined, and seroconversions due to natural infection versus COVID-19 vaccination were not differentiated (13). The ACTIVATE-2 trial in older Greek patients (*N *= 153) did report a protective effect of BCG vaccination (odds ratio [OR] 0.32; 95% CI, 0.13 to 0.79), but the trial endpoint combined test-confirmed and suspected COVID-19 cases (16). A trial in India (*N* = 495) reported a protective effect for suspected COVID-19 cases (OR, 0·38; 95% CI, 0.20 to 0.72) but not for test-confirmed cases (OR, 1.08; 95% CI, 0.54 to 2.14) (17). It has been hypothesized that childhood BCG vaccination and/or latent tuberculosis might modulate BCG-induced trained immunity (18). Childhood vaccination is common in South Africa, Greece, and India but not in the Netherlands. Latent tuberculosis is more prevalent in South Africa than in Greece (48.5% and 5.8% in the South African and Greek trial populations, respectively) (4, 13), and uncommon in the Netherlands. It therefore seems unlikely that these factors explain the divergent results of the Greek and Indian versus South African and Dutch trials, but ongoing trials in additional populations might shed more light on this debate in the future (19, 20). We think that it is more likely that endpoints based on suspected COVID-19 cases are insufficiently specific for SARS-CoV-2 infection and that the total number of endpoints in the Greek and Indian trials was too small to rule out chance (16, 17).

SARS-CoV-2 risk in HCWs increased with the proportion of work hours with patient contact. This suggests that the risk is mostly due to a high probability of exposure rather than a higher risk of infection once exposed. Interestingly, working in intensive or medium care unit was associated with a lower risk of infection than working in urgent care, which may be related to better infection control measures, including availability of personal protective equipment, in the former. Using antihypertensive medication was also consistently associated with higher infection risk. Patients diagnosed with high blood pressure have increased levels of angiotensin-converting enzyme 2 (ACE2), which acts as the entry receptor for SARS-CoV-2 (21, 22). Whether antihypertensive medication use has an additional effect is controversial: animal models have suggested an increase in ACE2 in the respiratory tract due to these medications but an increased SARS-CoV-2 infection risk after medication initiation has not been confirmed in humans (23, 24). Finally, our data confirmed that a substantial proportion of SARS-CoV-2 infections (14.1%) are asymptomatic and that HCWs are less likely to seek testing for asymptomatic or very mild infections than for mild or moderate infections.

The BCG-Corona trial was initiated in March 2020, early in the first epidemic wave in the Netherlands, and therefore captured SARS-CoV-2 exposures and infections in an immunologically naive population. This may explain why the mean acute infection episode duration was long (15.7 days) and a substantial proportion of infected individuals developed lingering symptoms (10% long-COVID plus an additional 4% long-term loss of smell and/or taste). Studies in the United Kingdom showed that median episode duration declined as the pandemic progressed, from a median of 11 days in 2020 (25), to 8 days during Delta dominance in 2021 and 5 days during Omicron dominance in the winter of 2021–2022 (26). The proportion of COVID-19 patients developing long-COVID varied widely between studies due to differences in definitions used. A large population-based cohort in Groningen province in the Netherlands reported a prevalence of 12.7% for the period March 2020 to August 2021 using 23 symptoms, including loss of smell and/or taste, and a total symptom duration of at least 90 days after the diagnosis (27).

The percentage of participants with SARS-CoV-2 seroconversion at month 3 (7.8% versus 2.8%; *P* = 0.006), as well as mean SARS-CoV-2 anti-S1 antibody concentrations (13.1 versus 4.3 IU/mL; *P* = 0.023), were higher in the BCG group than in the placebo group, but no differences were seen at month 6 or month 12. Mean SARS-CoV-2 anti-N antibody concentrations at month 12 (not assessed at month 3 and month 6) also did not differ between randomization groups. This suggested that BCG vaccination may enhance SARS-CoV-2 antibody production after SARS-CoV-2 infection but that the effect is short-lived. This finding is not robust, because the number of participants with a SARS-CoV-2 infection in the first 3 months of the study was small and the overall anti-S1 antibody concentrations at month 3 were low. However, additional evidence for BCG potentially acting as an adjuvant comes from human challenge studies in which participants received BCG (re)vaccination followed by influenza or COVID-19 vaccination (7, 28).

Important strengths of this trial are the large number and comprehensive nature of the endpoints. Anti-S1 antibodies are highly sensitive and specific for the presence of the SARS-CoV-2 Spike protein but are induced by both natural infection as well as COVID-19 vaccines (29, 30). Anti-N seropositivity is considered less sensitive and specific (29, 31), but it is currently the most reliable way to identify natural SARS-CoV-2 infections after COVID-19 vaccination. A recent RIVM study using the same assay that we used in this trial showed that anti-N seropositivity was 85% sensitive for mild infections and 67% sensitive for asymptomatic infections (32). While we may have missed some natural infections in the second period of the study, there were no differences between the randomization groups in the rate of COVID-19 vaccinations and proportion of participants vaccinated by the end of the study. We would therefore expect a similar number of natural infections to have been missed in each group. Additional strengths of the trial included the high diary app completion and retention rates. Furthermore, the reliability of participant-reported positive tests was high. The concordance of participant-reported positive test results with hospital laboratory data was 89% in the one hospital for which laboratory data were available (see Text S1 in the supplemental material). Negative test results, on the other hand, were substantially underreported. While these were not endpoints in any of our analyses, we did use them to rule out infection episodes potentially responsible for seroconversion. Another potential limitation was that we used two different methods of blood collection: clinician-performed venipuncture and participant-performed fingerprick sampling (33).

10.1128/mbio.00356-23.1 TEXT S1 Supplementary background and methods. Download Text S1, DOCX file, 0.1 MB. Copyright © 2023 Claus et al. 2023 Claus et al. https://creativecommons.org/licenses/by/4.0/ This content is distributed under the terms of the Creative Commons Attribution 4.0 International license.

In conclusion, BCG vaccination of HCWs did not reduce SARS-CoV-2 infections nor infection duration or severity (on a scale from asymptomatic to moderate). In the first 3 months after vaccination, BCG vaccination may enhance SARS-CoV-2 antibody production during SARS-CoV-2 infection, but this remains to be confirmed.

## MATERIALS AND METHODS

### Study design and population.

The BCG-Corona trial was a multicenter, double-blind, placebo-controlled randomized trial comparing BCG to placebo vaccination to prevent absenteeism and COVID-19-related endpoints. The study protocol was approved by the institutional review board of the University Medical Center (UMC) Utrecht, registered at clinicaltrials.gov (identifier NCT04328441), and published (34). The sample size was determined for the primary objective (absenteeism) by computer simulation. The primary results were also published and summarized in a supplementary text (12). Participants were doctors, nurses, paramedics, and support staff from 9 Dutch hospitals: three university hospitals that implemented in-hospital sampling (referred to as the core hospitals) and six hospitals (one university and five nonuniversity teaching hospitals) that did not. Participants were 18 years or older, were expected to be in direct contact with SARS-CoV-2-infected patients, and possessed a smartphone. The primary exclusion criteria were known allergy to BCG, active or latent Mycobacterium tuberculosis infection (as judged by the local Principal Investigator in each hospital), any other active infection, immunocompromised state, malignancy or lymphoma in the past 2 years, current or planned pregnancy, any vaccination in the past 4 weeks, having a hospital employment contract of less than 22 h per week, or expected work absence of at least 4 weeks.

### Study procedures.

After having obtained informed consent, participants were randomized to BCG or placebo (1:1) using a computer-generated dynamic randomization algorithm in random blocks of two, four, or six sequences, stratified by hospital. They received an intradermal injection in the left upper arm with either 0.1 mL of the Danish strain 1331 (Statens Serum Institut, Denmark), equivalent to 0.075 mg attenuated Mycobacterium bovis, or 0.1 mL of normal saline solution. Participants and study personnel conducting participant follow-up were blinded to treatment allocation. The study personnel preparing and administering the study vaccines and the data analysts were not blinded but could not influence treatment allocations or data collection.

At the randomization visit, participants completed an online baseline questionnaire (Research Online, Julius Center, UMC Utrecht, the Netherlands) and installed a diary application on their smartphone (Research Follow App, Your Research BV, Huizen, Netherlands). Participants were asked to report symptoms via the app daily, as well as SARS-CoV-2 exposures and test results and health care visits, including hospital admissions, weekly. The daily questionnaire was integrated into the weekly questionnaire after 6 months to improve user convenience, and COVID-19 vaccination questions were added after the start of the Dutch vaccination campaign on 6 January 2021. We used push notifications, e-mails, and phone calls to maximize adherence with app completion, and we terminated the app for all participants on 27 March 2021. Blood sampling continued until mid-June 2021, and we collected data on symptoms, positive SARS-CoV-2 tests, and COVID-19 vaccinations for the period between 27 March 2021 and the participant’s final sampling date via an online questionnaire (Formdesk, Innovero Software Solutions BV, Wassenaar, Netherlands) and e-mail.

Blood samples were collected in two sampling rounds, dividing the follow-up time into two potential seroconversion periods. Period 1 was the period between study vaccination and the first sampling round, after about 3 months for the core hospitals and about 6 months for the other hospitals. Period 2 was the period between the first and the second sampling rounds, about 12 months after study vaccination for all hospitals. Core hospital participants donated 10 mL of serum via in-hospital venipuncture, which was processed, frozen, and transported frozen to the Dutch National Institute for Public Health and the Environment (RIVM in Dutch). The other participants, as well as core hospital participants who missed their venipuncture visit, were asked to collect about 300 μL of peripheral capillary blood at home by fingerprick using a sampling kit that was sent to them by mail; they returned the sample by mail to the RIVM laboratory on the day of collection. The RIVM laboratory used an in-house magnetic immunoassay on a Luminex platform to determine the presence and concentrations of SARS-CoV-2-specific antibodies (Text S1) (32, 35, 36). At the end of period 1, only immunoglobulin G (IgG) antibodies against the S1 subunit of the SARS-CoV-2 Spike protein (anti-S1) were measured. At the end of period 2, both anti-S1 and IgG antibodies against the SARS-CoV-2 nucleocapsid protein (anti-N) were measured to enable differentiation between natural infections and COVID-19 vaccine-induced antibodies.

### Outcome and follow-up time definitions.

The presence of a SARS-CoV-2 infection was defined as a participant-reported positive test result and/or seroconversion, which in turn was defined as anti-S1 seropositivity at the end of period 1 or anti-S1 and anti-N seropositivity at the end of period 2 (Text S1). Participants with anti-N but no anti-S1 seropositivity at the end of period 2, and no corresponding positive test date, were classified as having “inconclusive episodes” and were excluded from most analyses except for some sensitivity analyses (Text S1). Participants who had less than 80% app completion without self-reported or serological evidence of an infection were also excluded because we could not be sure that they were true negatives.

Episode duration and severity were based on the diary app data (Text S1). The acute infection episode duration was defined as the number of consecutive days during which the participant reported symptoms, not including stand-alone loss of smell and/or taste or lingering symptoms (e.g., fatigue) after respiratory symptoms had ceased. Fever was defined as a temperature of 38°C or above. All other symptoms were reported on a scale of 0 to 5, with 0 for not present and 1 to 5 corresponding to increasing severity. Episode severity was categorized as asymptomatic, mild, or moderate, as defined by the World Health Organization (WHO) (37, 38). Additionally, we further subdivided the WHO-defined mild category into very mild and mild subcategories (Text S1). Separate variables were created for long-term loss of smell and/or taste and long-COVID (based on symptoms other than stand-alone loss of smell and/or taste). These were defined as continuing to report the respective symptoms for at least 60 days after the end of the acute infection episode.

The number of follow-up days was calculated as the number of days between vaccination and the first infection episode (the positive test date or the start date of the symptomatic period if no test date was available; for survival analyses) or the end of study (the month 12 sampling date or the last day of app completion if the participant did not provide a month 12 sample; for all other analyses). The app completion percentage was calculated for the period between vaccination and 27 March 2021 (Text S1).

### Statistical analyses.

Analyses were performed in R version 4.1.2 (PBC, Boston, MA, USA). We compared characteristics between randomization groups using the Wilcoxon rank sum test for continuous variables and chi-squared test for categorical variables. Endpoints were any SARS-CoV-2 infection or asymptomatic, mild (further subdivided into very mild and mild), or moderate infection; only the first infection per participant was included. The occurrence of endpoints in the randomization groups was modeled using (multinomial) logistic regression for cumulative incidence and Cox proportional hazards models for time to first event (the latter including infections that could be dated only). All models were first run unadjusted, followed by adjustment for potential confounders. Randomization group was forced into the multivariable models, and potential confounders were selected using a backward stepwise approach using the Akaike information criterion (*k* = 2.7) to select the final model. Sensitivity analyses were conducted assuming that the inconclusive episodes were either true episodes or not, or assuming that participants with less than 80% app completion and no reported or detected infection episode were true negatives.

### Data availability.

Individual participant data that underlie the results reported in this article will be made available after deidentification to investigators whose proposed use of the data has been approved by an independent review committee up to 5 years following publication. The study protocol will be available to anyone during this same time frame. Information regarding submitting proposals and accessing data may be found on https://dataverse.nl/.

## References

[1] Netea MG, Joosten LAB, Latz E, Mills KHG, Natoli G, Stunnenberg HG, O'Neill LAJ, Xavier RJ. 2016. Trained immunity: a program of innate immune memory in health and disease. Science 352:aaf1098. doi:10.1126/science.aaf1098. 27102489 PMC5087274

[2] Moorlag S, Arts RJW, van Crevel R, Netea MG. 2019. Non-specific effects of BCG vaccine on viral infections. Clin Microbiol Infect 25:1473–1478. doi:10.1016/j.cmi.2019.04.020. 31055165

[3] Higgins JPT, Soares-Weiser K, López-López JA, Kakourou A, Chaplin K, Christensen H, Martin NK, Sterne JAC, Reingold AL. 2016. Association of BCG, DTP, and measles containing vaccines with childhood mortality: systematic review. BMJ 355:i5170. doi:10.1136/bmj.i5170. 27737834 PMC5063034

[4] Giamarellos-Bourboulis EJ, Tsilika M, Moorlag S, Antonakos N, Kotsaki A, Domínguez-Andrés J, Kyriazopoulou E, Gkavogianni T, Adami M-E, Damoraki G, Koufargyris P, Karageorgos A, Bolanou A, Koenen H, van Crevel R, Droggiti D-I, Renieris G, Papadopoulos A, Netea MG. 2020. ACTIVATE: randomized clinical trial of BCG vaccination against infection in the elderly. Cell 183:315–323.e9. doi:10.1016/j.cell.2020.08.051. 32941801 PMC7462457

[5] Wardhana, Datau EA, Sultana A, Mandang VVV, Jim E. 2011. The efficacy of Bacillus Calmette-Guerin vaccinations for the prevention of acute upper respiratory tract infection in the elderly. Acta Medica Indones 43:185–190. 21979284

[6] Arts RJW, Moorlag SJCFM, Novakovic B, Li Y, Wang S-Y, Oosting M, Kumar V, Xavier RJ, Wijmenga C, Joosten LAB, Reusken CBEM, Benn CS, Aaby P, Koopmans MP, Stunnenberg HG, van Crevel R, Netea MG. 2018. BCG vaccination protects against experimental viral infection in humans through the induction of cytokines associated with trained immunity. Cell Host Microbe 23:89–100.e5. doi:10.1016/j.chom.2017.12.010. 29324233

[7] Leentjens J, Kox M, Stokman R, Gerretsen J, Diavatopoulos DA, van Crevel R, Rimmelzwaan GF, Pickkers P, Netea MG. 2015. BCG vaccination enhances the immunogenicity of subsequent influenza vaccination in healthy volunteers: a randomized, placebo-controlled pilot study. J Infect Dis 212:1930–1938. doi:10.1093/infdis/jiv332. 26071565

[8] Walk J, de Bree LCJ, Graumans W, Stoter R, van Gemert G-J, van de Vegte-Bolmer M, Teelen K, Hermsen CC, Arts RJW, Behet MC, Keramati F, Moorlag SJCFM, Yang ASP, van Crevel R, Aaby P, de Mast Q, van der Ven AJAM, Benn CS, Netea MG, Sauerwein RW. 2019. Outcomes of controlled human malaria infection after BCG vaccination. Nat Commun 10:874. doi:10.1038/s41467-019-08659-3. 30787276 PMC6382772

[9] Nguyen LH, Drew DA, Graham MS, Joshi AD, Guo C-G, Ma W, Mehta RS, Warner ET, Sikavi DR, Lo C-H, Kwon S, Song M, Mucci LA, Stampfer MJ, Willett WC, Eliassen AH, Hart JE, Chavarro JE, Rich-Edwards JW, Davies R, Capdevila J, Lee KA, Lochlainn MN, Varsavsky T, Sudre CH, Cardoso MJ, Wolf J, Spector TD, Ourselin S, Steves CJ, Chan AT, COronavirus Pandemic Epidemiology Consortium. 2020. Risk of COVID-19 among front-line health-care workers and the general community: a prospective cohort study. Lancet Public Health 5:e475–e483. doi:10.1016/S2468-2667(20)30164-X. 32745512 PMC7491202

[10] Rudberg A-S, Havervall S, Månberg A, Jernbom Falk A, Aguilera K, Ng H, Gabrielsson L, Salomonsson A-C, Hanke L, Murrell B, McInerney G, Olofsson J, Andersson E, Hellström C, Bayati S, Bergström S, Pin E, Sjöberg R, Tegel H, Hedhammar M, Phillipson M, Nilsson P, Hober S, Thålin C. 2020. SARS-CoV-2 exposure, symptoms and seroprevalence in healthcare workers in Sweden. Nat Commun 11:5064. doi:10.1038/s41467-020-18848-0. 33033249 PMC7544689

[11] Sikkens JJ, Buis DTP, Peters EJG, Dekker M, Schinkel M, Reijnders TDY, Schuurman AR, de Brabander J, Lavell AHA, Maas JJ, Koopsen J, Han AX, Russell CA, Schinkel J, Jonges M, Matamoros S, Jurriaans S, van Mansfeld R, Wiersinga WJ, Smulders YM, de Jong MD, Bomers MK. 2021. Serologic surveillance and phylogenetic analysis of SARS-CoV-2 infection among hospital health care workers. JAMA Netw Open 4:e2118554. doi:10.1001/jamanetworkopen.2021.18554. 34319354 PMC9437910

[12] Ten Doesschate T, van der Vaart TW, Debisarun PA, Taks E, Moorlag SJCFM, Paternotte N, Boersma WG, Kuiper VP, Roukens AHE, Rijnders BJA, Voss A, Veerman KM, Kerckhoffs APM, Oever JT, van Crevel R, van Nieuwkoop C, Lalmohamed A, van de Wijgert JHHM, Netea MG, Bonten MJM, van Werkhoven CH. 2022. Bacillus Calmette-Guérin vaccine to reduce healthcare worker absenteeism in COVID-19 pandemic, a randomized controlled trial. Clin Microbiol Infect 28:1278–1285. doi:10.1016/j.cmi.2022.04.009. 35489606 PMC9046133

[13] Upton CM, van Wijk RC, Mockeliunas L, Simonsson USH, McHarry K, van den Hoogen G, Muller C, von Delft A, van der Westhuizen H-M, van Crevel R, Walzl G, Baptista PM, Peter J, Diacon AH, BCG CORONA Consortium. 2022. Safety and efficacy of BCG re-vaccination in relation to COVID-19 morbidity in healthcare workers: a double-blind, randomised, controlled, phase 3 trial. EClinicalMedicine 48:101414. doi:10.1016/j.eclinm.2022.101414. 35582122 PMC9098089

[14] Moorlag SJCFM, Taks E, Ten Doesschate T, van der Vaart TW, Janssen AB, Müller L, Ostermann P, Dijkstra H, Lemmers H, Simonetti E, Mazur M, Schaal H, Ter Heine R, van de Veerdonk FL, Bleeker-Rovers CP, van Crevel R, Ten Oever J, de Jonge MI, Bonten MJ, van Werkhoven CH, Netea MG. 2022. Efficacy of BCG vaccination against respiratory tract infections in older adults during the coronavirus disease 2019 pandemic. Clin Infect Dis 75:e938–e946. doi:10.1093/cid/ciac182. 35247264 PMC8903481

[15] Koekenbier EL, Fohse K, van de Maat JS, Oosterheert JJ, van Nieuwkoop C, Hoogerwerf JJ, Grousch MP, van den Bosch MAAJ, van de Wijgert JHH, Netea MG, Rosendaal FR, Bonten MJM, Werkhoven CHHV, BCG-PRIME Study Group. 2023. Bacillus Calmette-Guérin vaccine for prevention of COVID-19 and other respiratory tract infections in older adults with comorbidities: a randomized controlled trial. Clin Microbiol Infect 2023 Feb 2:S1198-743X(23)00044-7. doi:10.1016/j.cmi.2023.01.019. PMC9892323 36736662

[16] Tsilika M, Taks E, Dolianitis K, Kotsaki A, Leventogiannis K, Damoulari C, Kostoula M, Paneta M, Adamis G, Papanikolaou I, Stamatelopoulos K, Bolanu A, Katsaros K, Delavinia C, Perdios I, Pandi A, Tsiakos K, Proios N, Kalogianni E, Delis I, Skliros E, Akinsoglou K, Perdikouli A, Poulakou G, Milionis H, Athanassopoulou E, Kalpaki E, Efstratiou L, Perraki V, Papadopoulos A, Netea MG, Giamarellos-Bourboulis EJ. 2022. ACTIVATE-2: a double-blind randomized trial of BCG vaccination against COVID-19 in individuals at risk. Front Immunol 13:873067. doi:10.3389/fimmu.2022.873067. 35865520 PMC9294453

[17] Sinha S, Ajayababu A, Thukral H, Gupta S, Guha SK, Basu A, Gupta G, Thakur P, Lingaiah R, Das BK, Singh UB, Singh R, Narang R, Bhowmik D, Wig N, Modak DC, Bandyopadhyay B, Chakrabarty B, Kapoor A, Tewari S, Prasad N, Hashim Z, Nath A, Kumari N, Goswami R, Pandey S, Pandey RM. 2022. Efficacy of Bacillus Calmette-Guérin (BCG) vaccination in reducing the incidence and severity of COVID-19 in high-risk population (BRIC): a phase III, multi-centre, quadruple-blind randomised control trial. Infect Dis Ther 11:2205–2217. doi:10.1007/s40121-022-00703-y. 36242739 PMC9568923

[18] Khan N, Downey J, Sanz J, Kaufmann E, Blankenhaus B, Pacis A, Pernet E, Ahmed E, Cardoso S, Nijnik A, Mazer B, Sassetti C, Behr MA, Soares MP, Barreiro LB, Divangahi M. 2020. M. tuberculosis reprograms hematopoietic stem cells to limit myelopoiesis and impair trained immunity. Cell 183:752–770.e22. doi:10.1016/j.cell.2020.09.062. 33125891 PMC7599081

[19] Pittet LF, Messina NL, Gardiner K, Orsini F, Abruzzo V, Bannister S, Bonten M, Campbell JL, Croda J, Dalcolmo M, Elia S, Germano S, Goodall C, Gwee A, Jamieson T, Jardim B, Kollmann TR, Guimarães Lacerda MV, Lee KJ, Legge D, Lucas M, Lynn DJ, McDonald E, Manning L, Munns CF, Perrett KP, Prat Aymerich C, Richmond P, Shann F, Sudbury E, Villanueva P, Wood NJ, Lieschke K, Subbarao K, Davidson A, Curtis N, BRACE Trial Consortium Group. 2021. BCG vaccination to reduce the impact of COVID-19 in healthcare workers: protocol for a randomised controlled trial (BRACE trial). BMJ Open 11:e052101. doi:10.1136/bmjopen-2021-052101. PMC8557250 34711598

[20] Madsen AMR, Schaltz-Buchholzer F, Benfield T, Bjerregaard-Andersen M, Dalgaard LS, Dam C, Ditlev SB, Faizi G, Johansen IS, Kofoed P-E, Kristensen GS, Loekkegaard ECL, Mogensen CB, Mohamed L, Ostenfeld A, Oedegaard ES, Soerensen MK, Wejse C, Jensen AKG, Nielsen S, Krause TG, Netea MG, Aaby P, Benn CS. 2020. Using BCG vaccine to enhance non-specific protection of health care workers during the COVID-19 pandemic: a structured summary of a study protocol for a randomised controlled trial in Denmark. Trials 21:799. doi:10.1186/s13063-020-04714-3. 32943115 PMC7495402

[21] Úri K, Fagyas M, Kertész A, Borbely A, Jenei C, Bene O, Csanadi Z, Paulus WJ, Edes I, Papp Z, Toth A, Lizanecz E. 2016. Circulating ACE2 activity correlates with cardiovascular disease development. J Renin Angiotensin Aldosterone Syst 17. doi:10.1177/1470320316668435. PMC5843890 27965422

[22] Li XC, Zhang J, Zhuo JL. 2017. The vasoprotective axes of the renin-angiotensin system: physiological relevance and therapeutic implications in cardiovascular, hypertensive and kidney diseases. Pharmacol Res 125:21–38. doi:10.1016/j.phrs.2017.06.005. 28619367 PMC5607101

[23] Mancia G, Rea F, Ludergnani M, Apolone G, Corrao G. 2020. Renin-angiotensin-aldosterone system blockers and the risk of Covid-19. N Engl J Med 382:2431–2440. doi:10.1056/NEJMoa2006923. 32356627 PMC7206933

[24] Reynolds HR, Adhikari S, Pulgarin C, Troxel AB, Iturrate E, Johnson SB, Hausvater A, Newman JD, Berger JS, Bangalore S, Katz SD, Fishman GI, Kunichoff D, Chen Y, Ogedegbe G, Hochman JS. 2020. Renin–angiotensin–aldosterone system inhibitors and risk of COVID-19. N Engl J Med 382:2441–2448. doi:10.1056/NEJMoa2008975. 32356628 PMC7206932

[25] Sudre CH, Murray B, Varsavsky T, Graham MS, Penfold RS, Bowyer RC, Pujol JC, Klaser K, Antonelli M, Canas LS, Molteni E, Modat M, Jorge Cardoso M, May A, Ganesh S, Davies R, Nguyen LH, Drew DA, Astley CM, Joshi AD, Merino J, Tsereteli N, Fall T, Gomez MF, Duncan EL, Menni C, Williams FMK, Franks PW, Chan AT, Wolf J, Ourselin S, Spector T, Steves CJ. 2021. Attributes and predictors of long COVID. Nat Med 27:626–631. doi:10.1038/s41591-021-01292-y. 33692530 PMC7611399

[26] Menni C, Valdes AM, Polidori L, Antonelli M, Penamakuri S, Nogal A, Louca P, May A, Figueiredo JC, Hu C, Molteni E, Canas L, Österdahl MF, Modat M, Sudre CH, Fox B, Hammers A, Wolf J, Capdevila J, Chan AT, David SP, Steves CJ, Ourselin S, Spector TD. 2022. Symptom prevalence, duration, and risk of hospital admission in individuals infected with SARS-CoV-2 during periods of omicron and delta variant dominance: a prospective observational study from the ZOE COVID Study. Lancet 399:1618–1624. doi:10.1016/S0140-6736(22)00327-0. 35397851 PMC8989396

[27] Ballering AV, van Zon SKR, Olde Hartman TC, Rosmalen JGM, Lifelines Corona Research Initiative. 2022. Persistence of somatic symptoms after COVID-19 in the Netherlands: an observational cohort study. Lancet 400:452–461. doi:10.1016/S0140-6736(22)01214-4. 35934007 PMC9352274

[28] Rakshit S, Adiga V, Ahmed A, Parthiban C, Kumar NC, Dwarkanath P, Shivalingaiah S, Rao S, D’Souza G, Dias M, Maguire TJA, Doores KJ, Zoodsma M, Geckin B, Dasgupta P, Babji S, van Meijgaarden KE, Joosten SA, Ottenhoff THM, Li Y, Netea MG, Stuart KD, De Rosa SA, McElrath MJ, Vyakarnam A. 2022. Evidence for the heterologous benefits of prior BCG vaccination on COVISHIELD^TM^ vaccine-induced immune responses in SARS-CoV-2 seronegative young Indian adults. Front Immunol 13:985938. doi:10.3389/fimmu.2022.985938. 36268023 PMC9577398

[29] Liu W, Liu L, Kou G, Zheng Y, Ding Y, Ni W, Wang Q, Tan L, Wu W, Tang S, Xiong Z, Zhang S. 2020. Evaluation of nucleocapsid and spike protein-based enzyme-linked immunosorbent assays for detecting antibodies against SARS-CoV-2. J Clin Microbiol 58:e00461-20. doi:10.1128/JCM.00461-20. 32229605 PMC7269413

[30] Solastie A, Virta C, Haveri A, Ekstrom N, Kantele A, Miettinen S, Lempainen J, Jalkanen P, Kakkola L, Dub T, Julkunen I, Melin M. 2021. A highly sensitive and specific SARS-CoV-2 spike- and nucleoprotein-based fluorescent multiplex immunoassay (FMIA) to measure IgG, IgA, and IgM class antibodies. Microbiol Spectr 9:e01131-21. doi:10.1128/Spectrum.01131-21. 34787485 PMC8597651

[31] Kontou PI, Braliou GG, Dimou NL, Nikolopoulos G, Bagos PG. 2020. Antibody tests in detecting SARS-CoV-2 infection: a meta-analysis. Diagnostics 10:319. doi:10.3390/diagnostics10050319. 32438677 PMC7278002

[32] van den Hoogen LL, Smits G, van Hagen CCE, Wong D, Vos ERA, van Boven M, de Melker HE, van Vliet J, Kuijer M, Woudstra L, Wijmenga-Monsuur AJ, GeurtsvanKessel CH, Stoof SP, Reukers D, Wijsman LA, Meijer A, Reusken CBEM, Rots NY, van der Klis FRM, van Binnendijk RS, den Hartog G. 2022. Seropositivity to nucleoprotein to detect mild and asymptomatic SARS-CoV-2 infections: a complementary tool to detect breakthrough infections after COVID-19 vaccination? Vaccine 40:2251–2257. doi:10.1016/j.vaccine.2022.03.009. 35287986 PMC8904156

[33] Triest D, Geebelen L, De Pauw R, et al. 2021. Performance of five rapid serological tests in mild-diseased subjects using finger prick blood for exposure assessment to SARS-CoV-2. J Clin Virol 142:104897. doi:10.1016/j.jcv.2021.104897. 34304089 PMC8282933

[34] Ten Doesschate T, Moorlag SJCFM, van der Vaart TW, Taks E, Debisarun P, Ten Oever J, Bleeker-Rovers CP, Verhagen PB, Lalmohamed A, Ter Heine R, van Crevel R, van de Wijgert J, Janssen AB, Bonten MJ, van Werkhoven CH, Netea MG, BCG-CORONA Study Team. 2020. Two randomized controlled trials of Bacillus Calmette-Guérin vaccination to reduce absenteeism among health care workers and hospital admission by elderly persons during the COVID-19 pandemic: a structured summary of the study protocols for two randomised controlled trials. Trials 21:481. doi:10.1186/s13063-020-04389-w. 32503602 PMC7273375

[35] den Hartog G, Schepp RM, Kuijer M, GeurtsvanKessel C, van Beek J, Rots N, Koopmans MPG, van der Klis FRM, van Binnendijk RS. 2020. SARS-CoV-2–specific antibody detection for seroepidemiology: a multiplex analysis approach accounting for accurate seroprevalence. J Infect Dis 222:1452–1461. doi:10.1093/infdis/jiaa479. 32766833 PMC7454740

[36] Vos ERA, van Boven M, den Hartog G, Backer JA, Klinkenberg D, van Hagen CCE, Boshuizen H, van Binnendijk RS, Mollema L, van der Klis FRM, de Melker HE. 2021. Associations between measures of social distancing and severe acute respiratory syndrome coronavirus 2 seropositivity: a nationwide population-based study in the Netherlands. Clin Infect Dis 73:2318–2321. doi:10.1093/cid/ciab264. 33772265 PMC8083720

[37] World Health Organization. 2021. COVID-19 clinical management: living guidance, 25 January 2021. https://apps.who.int/iris/handle/10665/338882. Accessed 6 Feb 2023.

[38] WHO Working Group on the Clinical Characterisation and Management of COVID-19 Infection. 2020. A minimal common outcome measure set for COVID-19 clinical research. Lancet Infect Dis 20:e192–e197. doi:10.1016/S1473-3099(20)30483-7. 32539990 PMC7292605

