# Supplementary material for: BCG Vaccination of Health Care Workers Does Not Reduce SARS-CoV-2 Infections nor Infection Severity or Duration: a Randomized Placebo-Controlled Trial
Source: mBio. 2023 Mar 28;14(2):e00356-23. doi: 10.1128/mbio.00356-23 (PMC10128007; doi:10.1128/mbio.00356-23)
Supplement: FIG S1 [file mbio.00356-23-s0002.docx]

**Figure S1: Completeness of smartphone diary application entries by A) all randomized participants (N=1511), and B) all participants that experienced an event (N=298)**


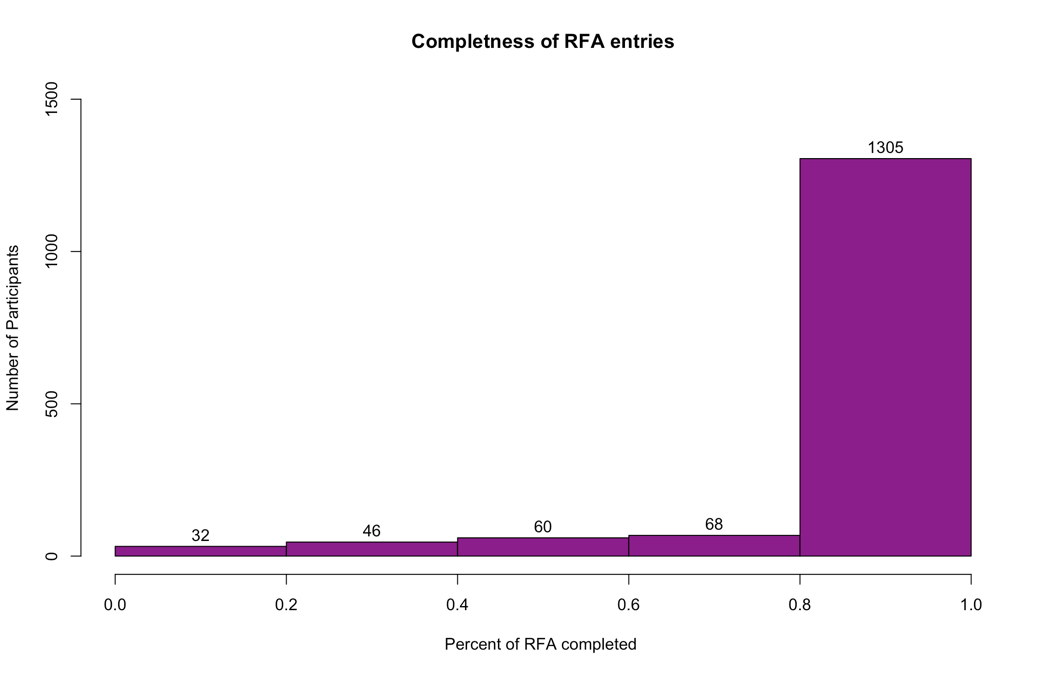


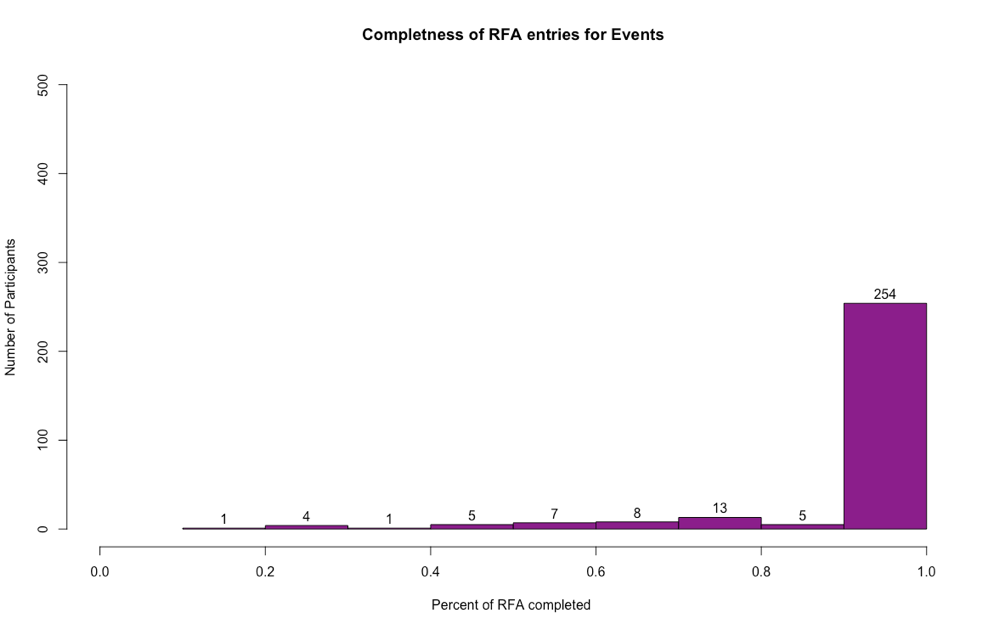


A) Completeness of diary entries for the entire randomized population (N=1511); completion percentages were not significantly different between randomization groups (p=0.283).

B) Completeness of diary entries for those that experienced an event (N=298). The 39 participants who did not complete at least 80% of expected diary entries were included in the analysis population because we did have clear evidence that they had an infection.
