# Supplementary material for: BCG Vaccination of Health Care Workers Does Not Reduce SARS-CoV-2 Infections nor Infection Severity or Duration: a Randomized Placebo-Controlled Trial
Source: mBio. 2023 Mar 28;14(2):e00356-23. doi: 10.1128/mbio.00356-23 (PMC10128007; doi:10.1128/mbio.00356-23)
Supplement: TABLE S1 [file mbio.00356-23-s0003.docx]

**Table S1A: Baseline characteristics of the randomized population**

| **Cells contain n (% of N) unless stated otherwise** | **BCG**  **(N=753)** | **Placebo**  **(N=758)** | **Total**  **(N=1,511)** | **p^1^** |
| --- | --- | --- | --- | --- |
| **Recruitment site** Radboud UMC  UMC Utrecht  Noordwest ZH Alkmaar  Haga ZH Den Haag  Canisius-Wilhelmina ZH Nijmegen  Sint Maartenskliniek Nijmegen  Leiden UMC  Jeroen Bosch ZH Den Bosch  Erasmus UMC | 209 (27.8)  193 (25.6)  158 (21.0)  50 (6.6)  38 (5.0)  31 (4.1)  28 (3.7)  25 (3.3)  21 (2.8) | 210 (27.7)  192 (25.3)  158 (20.8)  52 (6.9)  37 (4.9)  34 (4.5)  28 (3.7)  26 (3.4)  21 (2.8) | 419 (27.7)  385 (25.5)  316 (20.9)  102 (6.8)  75 (5.0)  65 (4.3)  56 (3.7)  51 (3.4)  42 (2.8) | 1.000 |
| **Age in years, mean (SD)^2^** | 41.31 (12.63) | 42.76 (12.73) | 42.04 (12.70) | **0.026** |
| **Female sex** | 572 (76.0) | 550 (72.6) | 1122 (74.3) | 0.146 |
| **# Additional household members, mean (SD)** | 2.00 (1.53) | 1.85 (1.41) | 1.93 (1.47) | 0.056 |
| **Smoking status** Current  Former  Never | 63 (8.4)  228 (30.3)  462 (61.4) | 60 (7.9)  208 (27.4)  490 (64.6) | 123 (8.1)  436 (28.9)  952 (63.0) | 0.407 |
| **Hospital department** Urgent care  Internal medicine^3^  Intensive/medium care  Other | 40 (5.3)  127 (16.9)  67 (8.9)  519 (68.9) | 47 (6.2)  109 (14.4)  72 (9.5)  530 (69.9) | 87 (5.8)  236 (15.6)  139 (9.2)  1049 (69.4) | 0.529 |
| **Job function** Doctor  Nurse  Paramedic  Support personnel | 172 (22.8)  363 (48.2)  123 (16.3)  95 (12.6) | 185 (24.4)  367 (48.4)  107 (14.1)  99 (13.1) | 357 (23.6)  730 (48.3)  230 (15.2)  194 (12.8) | 0.643 |
| **Scheduled to work** No  **on COVID-ward** Yes  Unknown | 226 (30.0)  481 (63.9)  46 (6.1) | 219 (28.9)  481 (63.5)  58 (7.7) | 445 (29.5)  962 (63.7)  104 (6.9) | 0.478 |
| **% work hours** 0-25  **with patient contact** 26-50  51-75  75+ | 111 (14.7)  120 (15.9)  131 (17.4)  391 (51.9) | 127 (16.8)  93 (12.3)  126 (16.6)  412 (54.4) | 238 (15.8)  213 (14.1)  257 (17.0)  803 (53.1) | 0.163 |
| **History of BCG vaccination** | 129 (17.1) | 127 (16.8) | 256 (16.9) | 0.899 |
| **Past tuberculosis test results^4^**  Tested negative  Tested positive (either or both)  Never tested  Unknown (both) | 504 (66.9)  58 (7.7)  181 (24.0)  10 (1.3) | 512 (67.5)  78 (10.3)  166 (21.9)  2 (0.3) | 1016 (67.2)  136 (9.0)  347 (23.0)  12 (0.8) | **0.030** |
| **Respiratory infection in winter 2019-2020**  No  Yes, with fever  Yes, no fever | 548 (72.8)  69 (9.2)  136 (18.1) | 542 (71.5)  58 (7.7)  158 (20.8) | 1090 (72.1)  127 (8.4)  294 (19.5) | 0.270 |
| **Influenza vaccination in winter 2020-2021**  Yes  No  Missing | 342 (45.4)  222 (29.5)  189 (25.1) | 355 (46.8)  206 (27.2)  197 (26.0) | 697 (46.1)  428 (28.3)  386 (25.5) | 0.610 |
| **Any other vaccination in past year^5^** | 85 (11.3) | 77 (10.2) | 162 (10.7) | 0.531 |
| **Current use of anti-hypertensive medication** | 49 (6.5) | 50 (6.6) | 99 (6.6) | 1.000 |
| **History of cardiovascular disease** | 15 (2.0) | 19 (2.5) | 34 (2.3) | 0.616 |
| **Current use of anti-diabetic medication** | 3 (0.4) | 6 (0.8) | 9 (0.6) | 0.510 |
| **History of asthma** | 54 (7.2) | 47 (6.2) | 101 (6.7) | 0.514 |
| **History of hay fever** | 229 (30.4) | 212 (28.0) | 441 (29.2) | 0.323 |
| **History of other pulmonary disease** | 14 (1.9) | 18 (2.4) | 32 (2.1) | 0.605 |
| **Any lung disease (previous three combined)^6^** | 254 (33.7) | 243 (32.1) | 497 (32.9) | 0.452 |
| **Positive SARS-CoV-2 test prior to baseline** | 1 (0.1) | 1 (0.11) | 2 (0.1) | 1.000 |
| **At least one dose COVID-19 vaccine during FU^7^** | 362 (43.3) | 335 (44.2) | 661 (43.7) | 0.736 |
| **COVID-19 vaccine type^7^**  Comirnaty  Spikevax  Vaxzevria  Jcovden  CureVac  Unknown  None | 203 (27.0)  69 (9.2)  44 (5.8)  7 (0.9)  0 (0.0)  3 (0.4)  427 (56.7) | 198 (26.1)  80 (10.6)  44 (5.8)  6 (0.8)  1 (0.1)  6 (0.8)  423 (55.8) | 401 (26.5)  149 (9.9)  88 (5.8)  13 (0.9)  1 (0.1)  9 (0.6)  850 (56.3) | 0.815 |

Abbreviations: FU=follow-up; SD=standard deviation; UMC=University Medical Center; ZH=ziekenhuis (hospital).

1. Chi-squared tests for categorical variables and Wilcoxon rank sum test for continuous variables.
2. The 1.45 years difference in mean age is statistically significant but we believe that it is not relevant in this context.
3. Internal medicine includes the pulmonology and infectious disease departments.
4. Tuberculosis tests include the Mantoux and/or TB QuantiFERON tests. The statistical difference between the BCG and placebo groups is for the unknown category only.
5. The following other vaccinations were reported: DTaP-IPV, hepatitis A, hepatitis B, yellow fever, typhoid, rabies, mumps-measles-rubella, meningococcal, pneumococcal, *Haemophilus influenzae* type B, Ebola, tick-borne encephalitis, human papillomavirus, and unknown.
6. “Any lung disease” includes asthma, hay fever, and any other pulmonary disease.
7. In the Netherlands, the SARS-CoV-2 vaccines available during the study period were Comirnaty (Pfizer/BioNTech, New York, NY, USA), Spikevax (Moderna Biotech, Cambridge, MA, USA), Vaxzevria (AstraZeneca AB, Sodertalje, Sweden), and Jcovden (Janssen Vaccines, Leiden, Netherlands). In addition, one participant received an experimental vaccine by CureVac N.V. in a clinical trial setting. This vaccine was never marketed due to insufficient efficacy.

**Table S1B: Baseline characteristics of core hospitals vs. other hospitals**

| **Cells contain n (% of N) unless stated otherwise** | **Core hospitals**  **(N=738)** | **Other hospitals**  **(N=571)** | **Total**  **(N=1,309)** | **p^1^** |
| --- | --- | --- | --- | --- |
| **Participated in sampling**  Round 1  Round 2 | 698 (94.6)  649 (87.9) | 437 (76.5)  454 (79.5) | 1135 (86.7)  1103 (84.3) | **<0.001**  **<0.001** |
| **Age in years, mean (SD)^2^** | 41.73 (13.06) | 43.48 (12.18) | 42.49 (12.71) | **0.013** |
| **Female sex** | 532 (72.1) | 442 (77.4) | 974 (74.4) | 0.034 |
| **# Additional household members, mean (SD)** | 1.89 (1.45) | 1.93 (1.32 | 1.91 (1.39) | 0.605 |
| **Smoking status** Current  Former  Never | 37 (5.0)  202 (27.4)  499 (67.6) | 58 (10.2)  191 (33.5)  322 (56.4) | 95 (7.3)  393 (30.0)  821 (62.7) | **<0.001** |
| **Hospital department** Urgent care  Internal medicine^3^  Intensive/medium care  Other | 26 (3.5)  95 (12.9)  73 (9.9)  544 (73.7) | 49 (8.6)  108 (18.9)  54 (9.5)  360 (63.0) | 75 (5.7)  203 (15.5)  127 (9.7)  904 (69.1) | **<0.001** |
| **Job function** Doctor  Nurse  Paramedic  Support personnel | 165 (22.4)  345 (46.7)  125 (16.9)  103 (14.0) | 129 (22.6)  300 (52.5)  79 (13.8)  63 (11.0) | 294 (22.5)  645 (49.3)  204 (15.6)  166 (12.7) | 0.095 |
| **Scheduled to work** No  **on COVID-ward** Yes  Unknown | 280 (37.9)  387 (52.4)  71 (69.6) | 106 (18.6)  449 (78.6)  16 (2.8) | 386 (29.5)  836 (63.9)  87 (6.7) | **<0.001** |
| **% work hours** 0-25  **with patient contact** 26-50  51-75  75+ | 145 (19.6)  125 (16.9)  150 (20.3)  318 (43.1) | 62 (10.9)  65 (11.4)  67 (11.7)  377 (66.0) | 207 (15.8)  190 (14.5)  217 (16.6)  695 (53.1) | **<0.001** |
| **History of BCG vaccination** | 145 (19.6) | 81 (14.2) | 226 (17.3) | 0.012 |
| **Past tuberculosis test results^4^**  Tested negative  Tested positive (either or both)  Never tested  Unknown (both) | 479 (64.9)  71 (9.6)  179 (24.3)  19 (1.2) | 410 (71.8)  51 (8.9)  107 (18.7)  3 (0.5) | 889 (67.9)  122 (9.3)  286 (21.8)  12 (0.9) | **0.035** |
| **Respiratory infection in winter 2019-2020**  No  Yes, with fever  Yes, no fever | 530 (71.8)  71 (9.6)  137 (18.6) | 414 (72.5)  42 (7.4)  115 (20.1) | 944 (72.1)  113 (8.6)  252 (19.3) | 0.309 |
| **Influenza vaccination in winter 2020-2021**  Yes  No  Missing | 401 (54.3)  214 (29.0)  123 (16.7) | 273 (47.8)  194 (34.0)  104 (18.2) | 674 (51.5)  408 (31.2)  227 (17.3) | 0.059 |
| **Any other vaccination in past year^5^** | 82 (11.1) | 54 (9.5) | 136 (10.4) | 0.378 |
| **Current use of anti-hypertensive medication** | 51 (6.9) | 39 (6.8) | 90 (6.9) | 1.000 |
| **History of cardiovascular disease** | 17 (2.3) | 13 (2.3) | 30 (2.3) | 1.000 |
| **Current use of anti-diabetic medication** | 4 (0.5) | 3 (0.5) | 7 (0.5) | 1.000 |
| **History of asthma** | 48 (6.5) | 43 (7.5) | 91 (7.0) | 0.539 |
| **History of hay fever** | 236 (32.0) | 145 (25.4) | 381 (29.1) | 0.011 |
| **History of other pulmonary disease** | 14 (1.9) | 15 (2.6) | 29 (2.2) | 0.484 |
| **Any lung disease (previous three combined)^6^** | 256 (34.7) | 175 (30.6) | 431 (32.9) | 0.138 |
| **Positive SARS-CoV-2 test prior to baseline** | 1 (0.1) | 0 (0.0) | 1 (0.1) | 0.898 |
| **At least one dose COVID-19 vaccine during FU^7^** | 285 (38.6) | 354 (62.0) | 639 (48.8) | **<0.001** |

Abbreviations: FU=follow-up; SD=standard deviation.

- - 1. Chi-squared tests for categorical variables and Wilcoxon rank sum test for continuous variables.
    2. We believe that this significant difference is not meaningful in this context.
    3. Internal medicine includes the pulmonology and infectious disease departments.
    4. Tuberculosis tests include the Mantoux and/or TB QuantiFERON tests. The statistical difference between the BCG and placebo groups is for the unknown category only.
    5. The following other vaccinations were reported: DTaP-IPV, hepatitis A, hepatitis B, yellow fever, typhoid, rabies, mumps-measles-rubella, meningococcal, pneumococcal, *Haemophilus influenzae* type B, Ebola, tick-borne encephalitis, human papillomavirus, and unknown.
    6. “Any lung disease” includes asthma, hay fever, and any other pulmonary disease.
    7. In the Netherlands, the SARS-CoV-2 vaccines available during the study period were Comirnaty (Pfizer/BioNTech, New York, NY, USA), Spikevax (Moderna Biotech, Cambridge, MA, USA), Vaxzevria (AstraZeneca AB, Sodertalje, Sweden), and Jcovden (Janssen Vaccines, Leiden, Netherlands). In addition, one participant received an experimental vaccine by CureVac N.V. in a clinical trial setting. This vaccine was never marketed due to insufficient efficacy.
