# Supplementary material for: BCG Vaccination of Health Care Workers Does Not Reduce SARS-CoV-2 Infections nor Infection Severity or Duration: a Randomized Placebo-Controlled Trial
Source: mBio. 2023 Mar 28;14(2):e00356-23. doi: 10.1128/mbio.00356-23 (PMC10128007; doi:10.1128/mbio.00356-23)
Supplement: FIG S2 [file mbio.00356-23-s0004.docx]

**Figure S2: SARS-CoV-2 infections over calendar time in the study population for all infections (A), by infection severity (WHO definitions with subcategories) (B), by recruitment site (C), and in the Netherlands as a whole (D)**

**
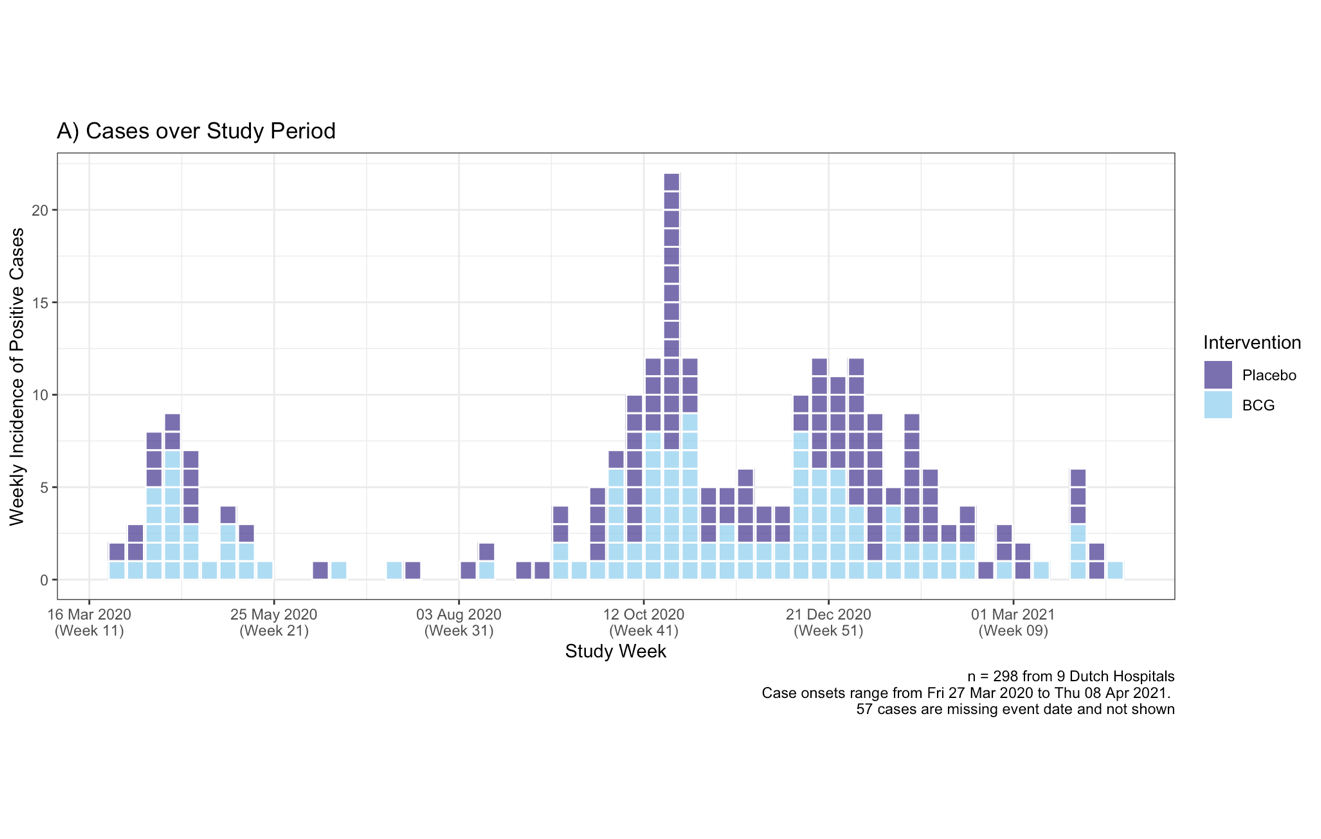
**


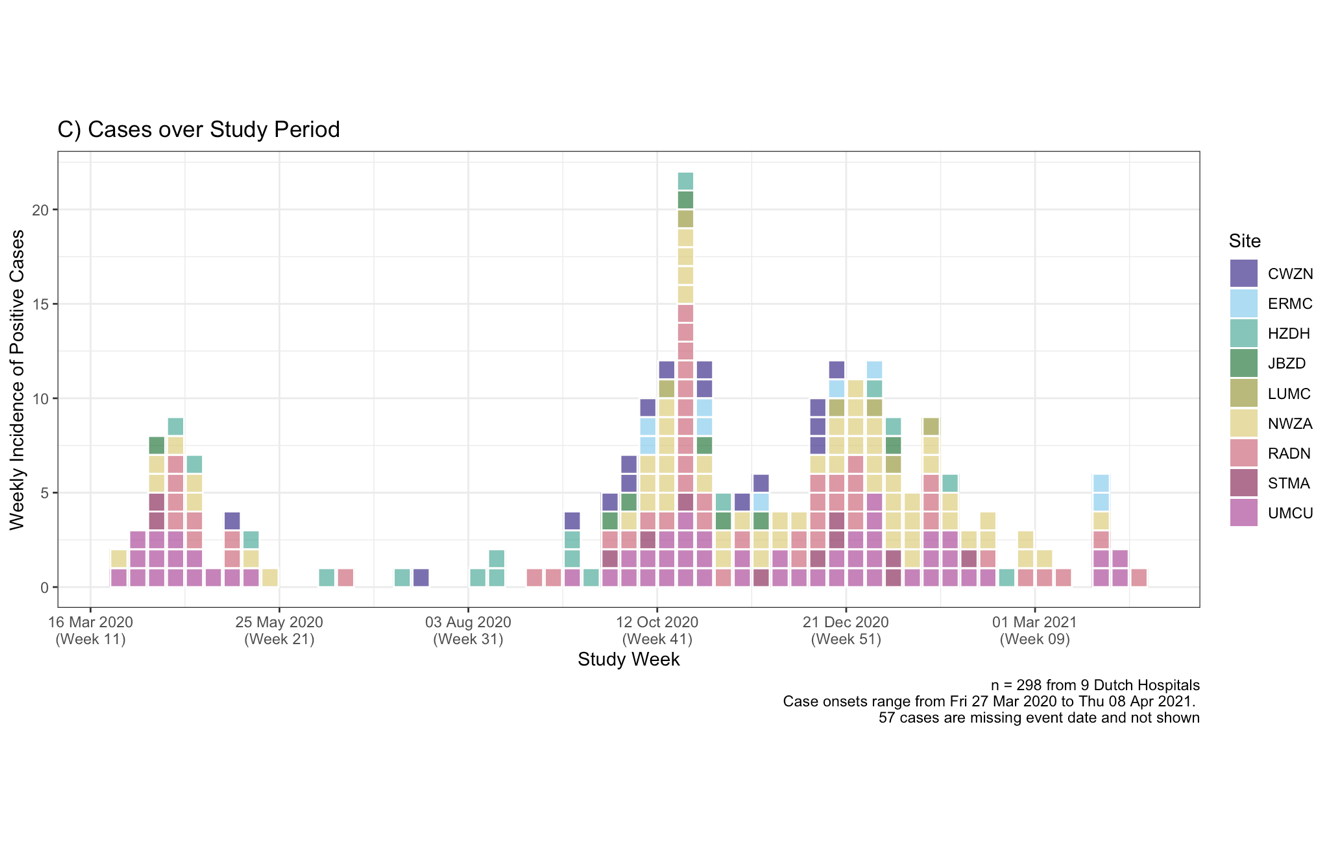
**
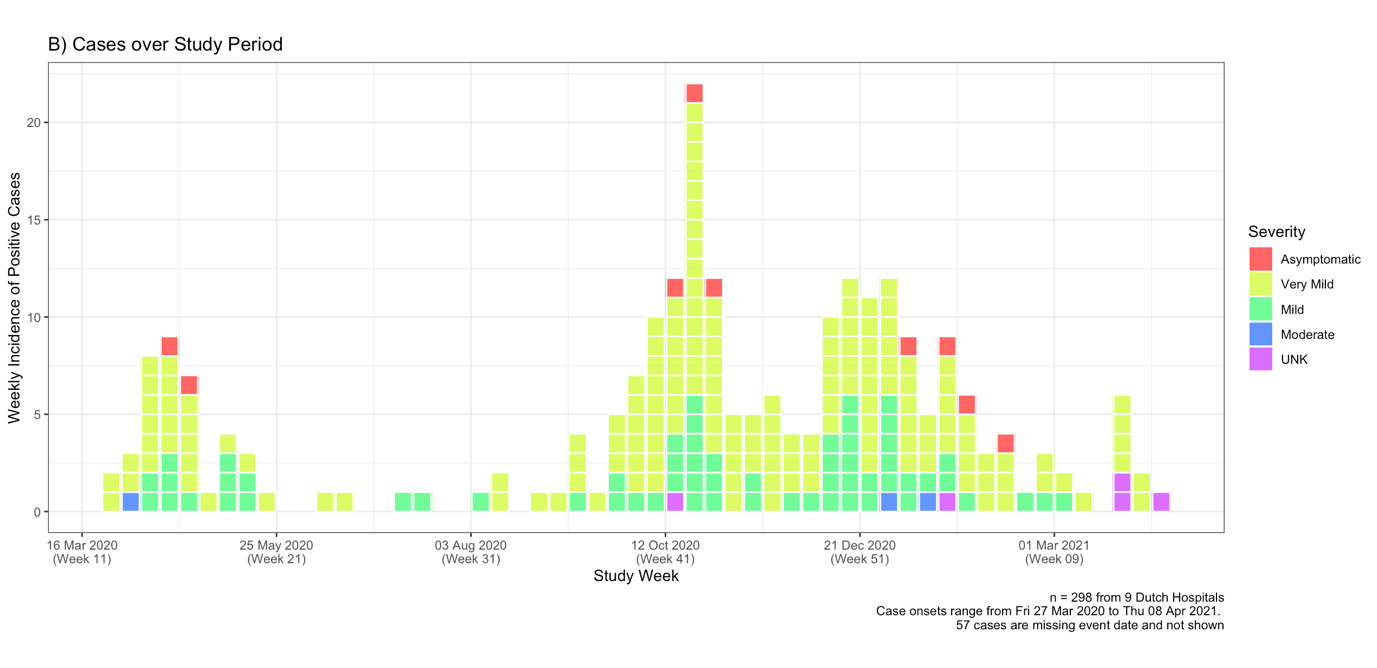
**

D)

**
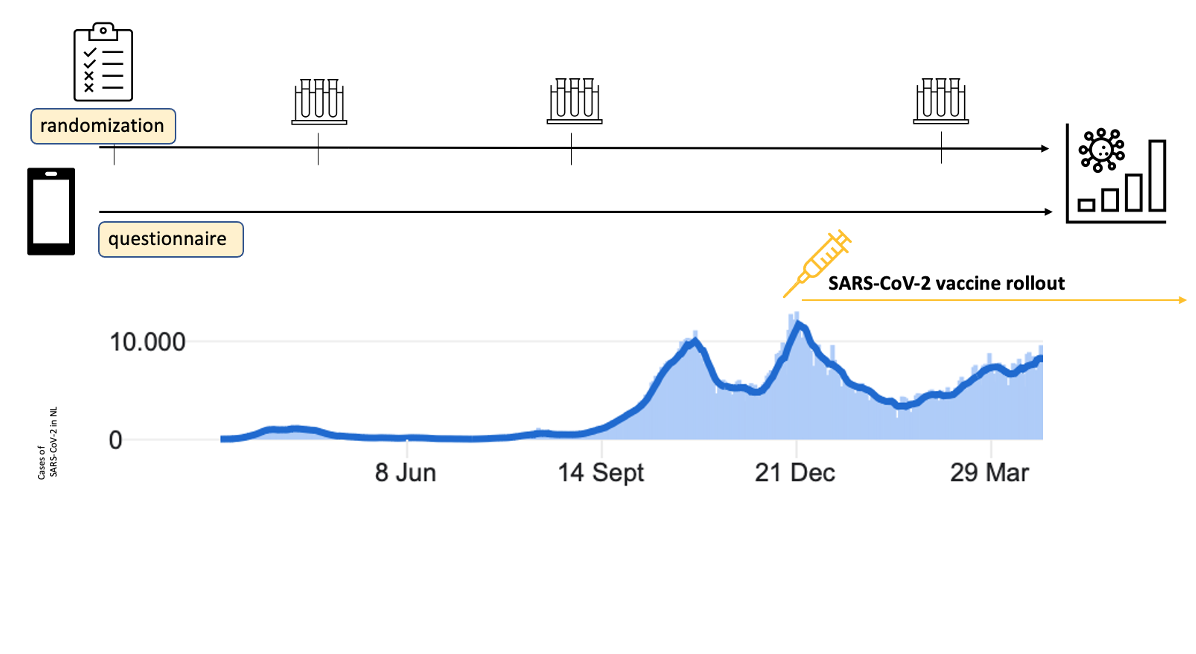
**

**Panel D:** Incidence of SARS-CoV-2 cases in the Netherlands during the study follow-up period. SARS-CoV-2 infections over calendar time in the study population mirrored epidemic waves in the Netherlands except for the March-May 2020 wave. Infections in the general public were under detected during that wave because public testing was not yet widely available. In contrast, healthcare workers could get tested during that wave in the hospitals where they worked.

Data sources:

1. The Corona dashboard of the Dutch National Institute for Health and Environment (RIVM): <https://coronadashboard.rijksoverheid.nl/>
2. COVID-19 Data Repository by the Center for Systems Science and Engineering at Johns Hopkins University (Dong E, Du H, Gardner L. An interactive web-based dashboard to track COVID-19 in real time. *Lancet Inf Dis* 2020; 20(5):533-534. doi: 10.1016/S1473-3099(20)30120-1).
