# Supplementary material for: BCG Vaccination of Health Care Workers Does Not Reduce SARS-CoV-2 Infections nor Infection Severity or Duration: a Randomized Placebo-Controlled Trial
Source: mBio. 2023 Mar 28;14(2):e00356-23. doi: 10.1128/mbio.00356-23 (PMC10128007; doi:10.1128/mbio.00356-23)
Supplement: TABLE S2 [file mbio.00356-23-s0005.docx]

**Table S2: Additional outcomes during follow-up in the analysis population^1^**

| **N=participants** | **BCG**  **(N=665)** | **Placebo**  **(N=644)** | **Total**  **(N=1,309)** | **p^2^** |
| --- | --- | --- | --- | --- |
| Peron-years at risk | 597.08 | 585.49 | 1,182.57 |  |
| Incidence rate  Incidence rate ratio (95% CI) | 0.25 | 0.26 | 0.95 (0.76, 1.21) | 0.732 |
| Cumulative incidence | 0.22 | 0.23 | 0.23 | 0.608 |
| Anti-S1 antibodies present in round 2^3,4^, n (%) | 271 (49.2) | 275 (49.8) | 546 (49.5) | 0.880 |
| Anti-N antibodies present in round 2^4^, n (%) | 75 (13.6) | 88 (16.0) | 163 (14.8) | 0.315 |
| **N=episodes** | **BCG**  **(N=147)** | **Placebo**  **(N=151)** | **Total**  **(N=298)** | **p^2^** |
| Period that episode occurred in, n (%):  Period 1  Period 2 (or period 1 if no round 1 done)  Unknown | 57 (38.8)  89 (60.5)  1 (0.7) | 34 (22.5)  114 (75.5)  3 (2.0) | 91 (30.5)  203 (68.1)  4 (1.3) | **0.007** |
| Seroconversion status after episode, n (%):  Did not seroconvert  Episode in period 1, S+ in round 1  Episode in period (1+)2, S+N+ in round 2  Episode in period (1+)2, S+N- in round 2  Episode in period (1+)2, S-N+ in round 2  Unknown (no serology done or unknown period) | 8 (5.4)  46 (31.3)  57 (38.8)  9 (6.1)  0 (0.0)  27 (18.4) | 16 (10.6)  23 (15.2)  69 (45.7)  21 (13.9)  2 (1.3)  20 (13.2) | 24 (8.1)  69 (23.2)  126 (42.3)  30 (10.1)  2 (0.7)  47 (15.8) | **0.002** |
| Fever during episode, n (%)  Unknown | 50 (34.0)  3 (2.0) | 46 (30.5)  3 (2.0) | 96 (32.2)  6 (2.0) | 0.803 |
| Mean fever duration in days (SD) | 1.86 (3.30) | 2.20 (4.62) | 2.03 (4.02) | 0.477 |
| Dyspnea level,^5^ n (%):  0  1-3  4-5  Unknown/missing | 101 (68.7)  28 (19.0)  16 (10.9)  2 (1.4) | 107 (70.9)  29 (19.2)  12 (7.9)  3 (2.0) | 208 (69.8)  57 (19.1)  28 (9.4)  5 (1.7) | 0.823 |
| Mean dyspnea duration in days (SD) | 2.86 (7.99) | 3.40 (10.65) | 3.13 (9.43) | 0.627 |
| Other respiratory symptoms level,^5,6^ n (%):  0  1-3  4-5  Unknown/missing | 27 (18.4)  59 (40.1)  49 (33.3)  12 (8.2) | 29 (19.2)  62 (41.1)  49 (32.5)  11 (7.3) | 56 (18.8)  121 (40.6)  98 (32.9)  23 (7.7) | 0.987 |
| Mean respiratory symptoms duration in days (SD) | 8.70 (8.92) | 9.24 (9.92) | 8.98 (9.44) | 0.644 |
| Non-respiratory symptoms level,^6^ n (%):  0  1-3  4-5  Unknown/missing | 32 (21.8)  31 (21.1)  73 (49.7)  11 (7.5) | 32 (21.2)  29 (19.2)  80 (53.0)  10 (6.6) | 64 (21.5)  60 (20.1)  153 (51.3)  21 (7.0) | 0.944 |
| Total symptoms duration in weeks, n (%)  0 days (asymptomatic)  Up to 1 week  1-2 weeks  2-3 weeks  3-4 weeks  More than 4 weeks  Unknown (including ongoing at end of study) | 23 (15.6)  16 (10.9)  35 (23.8)  24 (16.3)  9 (6.1)  22 (15.0)  18 (12.2) | 19 (12.6)  28 (18.5)  29 (19.2)  21 (13.9)  12 (7.9)  27 (17.9)  15 (9.9) | 42 (14.1)  44 (15.1)  64 (21.5)  45 (15.1)  21 (7.0)  49 (16.4)  33 (11.1) | 0.472 |

Abbreviations: SD=standard deviation.

1. The main outcomes are shown in Table 2 of the manuscript.
2. Chi-square tests for categorical variables and Wilcoxon rank sum test for means.
3. Includes the participants who had received at least one dose of a COVID-19 vaccine. They were equally distributed among the randomization groups (Table 1 of the manuscript).
4. The denominators are 551 (665 minus 114 who did not provide a M12 sample) in the BCG group, 552 (644 minus 92) in the placebo group, and 1,103 in the total analysis population.
5. Highest level reported by the participant during the entire infection episode.
6. Does not include dyspnea (see separate mean duration for dyspnea) or loss of smell and/or taste.
