# Supplementary material for: BCG Vaccination of Health Care Workers Does Not Reduce SARS-CoV-2 Infections nor Infection Severity or Duration: a Randomized Placebo-Controlled Trial
Source: mBio. 2023 Mar 28;14(2):e00356-23. doi: 10.1128/mbio.00356-23 (PMC10128007; doi:10.1128/mbio.00356-23)
Supplement: FIG S3 [file mbio.00356-23-s0006.docx]

**Figure S3:** **Infection episode duration distributions by randomization group**


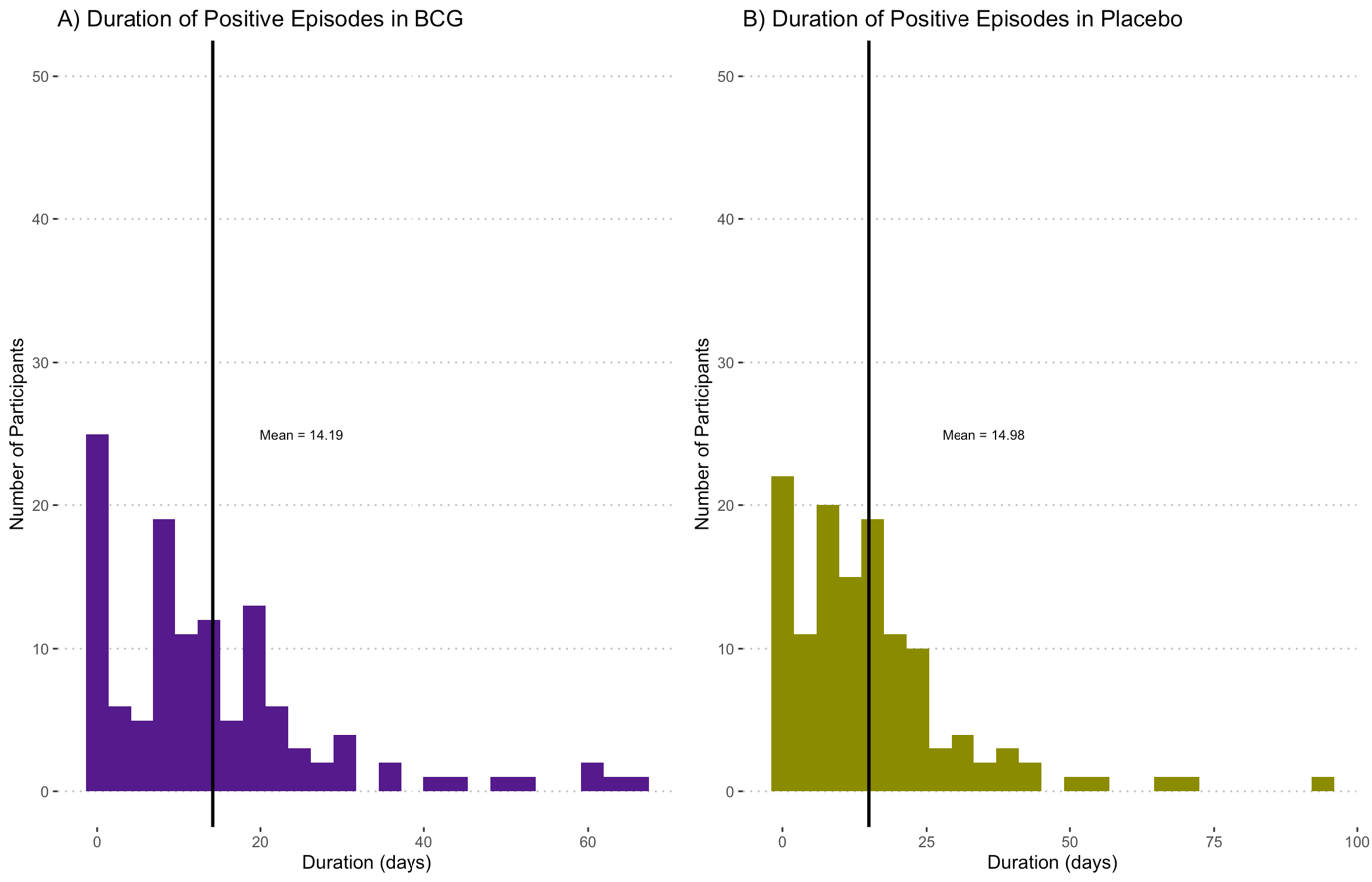


1. N=248 infection episodes: 49 episodes with unknown or ongoing duration at the end of study, and one outlier with duration of 398 are not shown (p=0.724).
2. A non-parametric test (Wilcoxon) was used to compare the mean duration between the groups. For the calculation of acute episode duration cases of long COVID-19 and episodes with unknow or ongoing duration were removed; N=189 and the difference in mean duration remained non-significant (0.890) (Table 2)
