# Supplementary material for: BCG Vaccination of Health Care Workers Does Not Reduce SARS-CoV-2 Infections nor Infection Severity or Duration: a Randomized Placebo-Controlled Trial
Source: mBio. 2023 Mar 28;14(2):e00356-23. doi: 10.1128/mbio.00356-23 (PMC10128007; doi:10.1128/mbio.00356-23)
Supplement: TABLE S3 [file mbio.00356-23-s0007.docx]

**Table S3: Univariable regression models with SARS-CoV-2 infection as outcome**

| **Covariate** | **Logistic regression** | | **Cox regression** | |
| --- | --- | --- | --- | --- |
|  | **OR (95% CI)** | **p** | **HR (95% CI)** | **p** |
| Age in years | 0.99 (0.98, 1.00) | **0.005** | 0.99 (0.98, 1.00) | **0.005** |
| Female sex | 1.34 (0.99, 1.84) | 0.065 | 1.32 (0.97, 1.80) | 0.075 |
| Additional household member | 1.06 (0.97, 1.16) | 0.217 | 1.08 (0.99, 1.17) | 0.101 |
| Smoking status  Current  Former  Never | --  1.19 (0.71, 2.07)  0.98 (0.59, 1.66) | --  0.531  0.924 | --  1.27 (0.75, 2.13)  0.99 (0.60, 1.63) | --  0.374  0.953 |
| Hospital department  Urgent care  Internal medicine^1^  Intensive/Medium care  Other | --  1.21 (0.68, 2.20)  0.60 (0.31, 1.18)  0.68 (0.40, 1.17) | --  0.520  0.135  0.146 | --  1.18 (0.68, 2.03)  0.45 (0.22, 0.91)  0.71 (0.43, 1.17) | --  0.557  **0.027**  0.176 |
| Job function  Doctor  Nurse  Paramedic  Support staff | --  1.95 (1.38, 2.80)  1.29 (0.81, 2.04)  1.18 (0.71, 1.93) | --  **<0.001**  0.281  0.522 | --  1.81 (1.27, 2.58)  1.26 (0.79, 2.00)  1.21 (0.74, 1.99) | --  **0.001**  0.335  0.452 |
| Scheduled to work on COVID-ward  No  Yes  Unknown | --  1.98 (1.45, 2.74)  1.55 (0.85, 2.73) | --  **<0.001**  0.139 | --  1.75 (1.27, 2.39)  1.47 (0.84, 2.58) | --  **<0.001**  0.179 |
| % work hours with patient contact:  0-25  26-50  51-75  75+ | --  1.68 (0.98, 2.93)  1.67 (0.99, 2.87)  2.68 (1.75, 4.25) | --  0.061  0.058  **<0.001** | --  1.69 (0.97, 2.92)  1.68 (0.98, 2.88)  2.37 (1.50, 3.75) | --  0.063  0.059  **<0.001** |
| History of BCG vaccination | 1.14 (0.81, 1.59) | 0.428 | 1.12 (0.81, 1.55) | 0.496 |
| Past tuberculosis test results^2^  Negative result  Positive result  Never Done  Unknown | --  1.18 (0.75, 1.82)  1.27 (0.93, 1.72)  0.73 (0.11, 2.78) | --  0.454  0.133  0.681 | --  1.10 (0.72, 1.69)  1.06 (0.78, 1.45)  0.90 (0.22, 3.64) | --  0.697  0.697  0.886 |
| Current use of anti-hypertensive medication | 1.50 (0.92, 2.38) | 0.092 | 1.53 (1.00, 2.33) | **0.049** |
| History of cardiovascular disease | 1.24 (0.51, 2.71) | 0.607 | 1.13 (0.50, 2.53) | 0.775 |
| Current use of anti-diabetic medication | 1.36 (0.19, 6.34) | 0.714 | 1.50 (0.37, 6.04) | 0.567 |
| History of asthma | 1.09 (0.65, 1.76) | 0.739 | 1.21 (0.77, 1.91) | 0.414 |
| History of hay fever | 1.19 (0.89, 1.57) | 0.231 | 1.16 (0.89, 1.53) | 0.272 |
| History of other pulmonary disease^3^ | 1.54 (0.66, 3.33) | 0.286 | 1.43 (0.67, 3.03) | 0.354 |
| Any Lung Disease^4^ | 1.21 (0.92, 1.58) | 0.166 | 1.19 (0.92, 1.55) | 0.188 |
| BCG vaccination^5^  Never  Past  Trial  Past and Trial | --  0.86 (0.51, 1.40)  0.84 (0.63, 1.12)  1.24 (0.78, 1.92) | --  0.563  0.230  0.352 | --  0.89 (0.55, 1.45)  0.85 (0.64, 1.12)  1.17 (0.76, 1.80) | --  0.636  0.249  0.469 |

Abbreviations: OR=odds ratio; HR=hazards ratio; 95% CI= 95% confidence interval.

1. Internal medicine includes the pulmonology and infectious disease departments
2. Tuberculosis tests include the Mantoux and/or TB QuantiFERON tests. The statistical difference between the BCG and placebo groups is for the unknown category only.
3. This does not include history of asthma or hay fever.
4. “Any lung disease” includes asthma, hay fever, and any other pulmonary disease.
5. Summary variable for participants that received a BCG vaccination is the past, in the trial, both, or never.
