# Supplementary material for: BCG Vaccination of Health Care Workers Does Not Reduce SARS-CoV-2 Infections nor Infection Severity or Duration: a Randomized Placebo-Controlled Trial
Source: mBio. 2023 Mar 28;14(2):e00356-23. doi: 10.1128/mbio.00356-23 (PMC10128007; doi:10.1128/mbio.00356-23)
Supplement: TABLE S4 [file mbio.00356-23-s0008.docx]

**Table S4: Multinomial logistic regression model with infection severity as the outcome^1^**

| **Covariates** | **Outcome categories** | **OR (95% CI)** | **p** |
| --- | --- | --- | --- |
| BCG versus placebo | No infection  Asymptomatic  Very mild  Mild  Moderate | --  1.13 (0.60, 2.11)  0.87 (0.62, 1.22)  0.72 (0.43, 1.21)  0.64 (0.05, 8.24) | --  0.711  0.416  0.230  0.729 |
| Age per year | No infection  Asymptomatic  Very mild  Mild  Moderate | --  0.99 (0.96, 1.02)  0.97 (0.96, 0.99)  1.00 (0.98, 1.02)  1.02 (0.89, 1.19) | --  0.390  **<0.001**  0.892  0.722 |
| Working in COVID-ward: yes vs. no | No infection  Asymptomatic  Very mild  Mild  Moderate | --  1.73 (0.73, 4.12)  1.51 (0.88, 2.07)  2.83 (1.34, 5.99)  0.91 (0.07,11.74) | --  0.217  0.171  **0.006**  0.941 |
| Working in COVID-ward: unknown vs. no | No infection  Asymptomatic  Very mild  Mild  Moderate | --  1.73 (0.43, 6.88)  1.31 (0.64, 2.70)  1.46 (0.38, 5.26)  Could not be estimated^2^ | --  0.439  0.461  0.582 |
| Hospital department: internal medicine vs. urgent care | No infection  Asymptomatic  Very mild  Mild  Moderate | --  0.92 (0.26, 3.25)  0.99 (0.50, 1.96)  7.86 (0.99, 62.2)  Could not be estimated^2^ | --  0.899  0.973  0.051 |
| Hospital department: intensive/medium care vs. urgent care | No infection  Asymptomatic  Very mild  Mild  Moderate | --  0.87 (0.24, 3.13)  0.19 (0.07, 0.53)  3.62 (0.43, 30.5)  Could not be estimated^2^ | --  0.830  **0.001**  0.236 |
| Hospital department: other vs. urgent care | No infection  Asymptomatic  Very mild  Mild  Moderate | --  0.56 (0.18, 1.77)  0.63 (0.34, 1.18)  4.42 (0.58, 33.4)  Could not be estimated^2^ | --  0.326  0.151  0.150 |
| Hospital function: nurse vs. doctor | No infection  Asymptomatic  Very mild  Mild  Moderate | --  1.94 (0.82, 4.63)  1.87 (1.19, 2.94)  3.87 (1.51, 8.89)  Could not be estimated^2^ | --  0.132  **0.007**  **0.004** |
| Hospital function: paramedic vs. doctor | No infection  Asymptomatic  Very mild  Mild  Moderate | --  1.08 (0.30, 3.86)  1.53 (0.86, 2.74)  2.02 (0.65, 6.28)  Could not be estimated^2^ | --  0.907  0.151  0.222 |
| Hospital function: support staff vs. doctor | No infection  Asymptomatic  Very Mild  Mild  Moderate | --  1.90 (0.56, 6.45)  0.99 (0.48, 2.02)  3.97 (1.32, 11.92)  Could not be estimated^2^ | --  0.301  0.978  **0.014** |
| Past BCG vaccination | No infection  Asymptomatic  Very mild  Mild  Moderate | --  1.83 (0.81, 4.13)  1.85 (1.16, 2.95)  1.27 (0.61, 2.66)  Could not be estimated^2^ | --  0.145  **0.009**  0.523 |
| Current use of hypertension medication | No infection  Asymptomatic  Very mild  Mild  Moderate | --  1.24 (0.35, 4.34)  2.06 (1.10, 3.84)  1.46 (0.55, 3.99)  36.42 (2.18, 609.30) | --  0.740  **0.024**  0.443  **0.012** |

Abbreviations: OR=odds ratio; 95% CI= 95% confidence interval.

1. N=1,309 participants and 298 endpoints with cumulative asymptomatic, very mild, mild, or moderate SARS-CoV-2 infections as endpoints. In addition to the variables listed in this table, the following variables were considered for inclusion in the model: recruitment site, enrolment week, sex, additional household members, percentage of work hours in contact with patients, ever having tested positive for tuberculosis, smoking status, current use of antidiabetic medication, history of pulmonary disease, and history of cardiovascular disease.
2. Could not be estimated because there were only 3 moderate cases in total.
