# Supplementary material for: BCG Vaccination of Health Care Workers Does Not Reduce SARS-CoV-2 Infections nor Infection Severity or Duration: a Randomized Placebo-Controlled Trial
Source: mBio. 2023 Mar 28;14(2):e00356-23. doi: 10.1128/mbio.00356-23 (PMC10128007; doi:10.1128/mbio.00356-23)
Supplement: TABLE S5 [file mbio.00356-23-s0009.docx]

**Table S5: Sensitivity analysis adding back in the participants with less than 80% diary app completion and no evidence of infection, assuming that they never had an infection**

1. **Logistic regression models (cumulative incidence)**

| **BCG vs placebo** | | **Analysis population^1^**  N=1,309  298 episodes | **Sensitivity analysis population^2^**  N=1,475  298 episodes |
| --- | --- | --- | --- |
|  |  | **OR (95% CI); p** | **OR (95% CI); p** |
| **Unadjusted** | |  |  |
| All episodes | | 0.93 (0.72, 1.20); 0.563 | 0.98 (0.76, 1.27); 0.887 |
| Participant-reported episodes only | | 0.89 (0.66, 1.18); 0.413 | 0.94 (0.71, 1.25); 0.666 |
| WHO Asymptomatic episodes only | | 1.15 (0.62, 2.16); 0.654 | 1.22 (0.66, 2.29); 0.527 |
| WHO Mild episodes only | | 0.89 (0.66, 1.18); 0.413 | 0.94 (0.71,1.25); 0.666 |
| Subcategory very mild episodes only | | 0.94 (0.67, 1.31); 0.715 | 1.00 (0.72, 1.38); 0.982 |
| Subcategory mild episodes only | | 0.76 (0.45, 1.27); 0.291 | 0.80 (0.47, 1.34); 0.401 |
| WHO Moderate episodes only | | 0.48 (0.02, 4.98); 0.545 | 0.50 (0.02, 5.28); 0.577 |
| **Adjusted for site and enrolment week** | |  |  |
| All episodes | | 0.93 (0.72, 1.20); 0.577 | 0.98 (0.76, 1.26); 0.869 |
| Participant-reported episodes only | | 0.88 (0.66, 1.18); 0.395 | 0.93 (0.69,1.24); 0.606 |
| **Adjusted for age and sex** | |  |  |
| All episodes | | 0.90 (0.70, 1.17); 0.443 | 0.96 (0.74, 1.24); 0.762 |
| Participant-reported episodes only | | 0.86 (0.64, 1.15); 0.304 | 0.91 (0.69, 1.22); 0.539 |
| **Multivariable (backward selection)^3^** | |  |  |
| All episodes | | 0.85 (0.65, 1.12); 0.249 | 0.92 (0.71, 1.20); 0.541 |
| Participant-reported episodes only | | 0.81 (0.59, 1.09); 0.161 | 0.87 (0.65, 1.17); 0.353 |
| **Multinomial** | |  |  |
| Unadjusted  All episodes | WHO Asymptomatic  WHO Mild  WHO Moderate | 1.15 (0.62, 2.14); 0.655  0.89 (0.66, 1.18); 0.413  0.48 (0.04, 5.27); 0.545 | 1.22 (0.66, 2.27); 0.527  0.94 (0.71, 1.25); 0.666  0.50 (0.05, 5.58); 0.577 |
|  | Asymptomatic  Very mild  Mild  Moderate | 1.15 (0.62, 2.14); 0.655  0.94 (0.68, 1.31); 0.715  0.76 (0.45, 1.27); 0.292  0.48 (0.04, 5.28); 0.546 | 1.22 (0.66, 2.27); 0.527  1.00 (0.72, 1.38); 0.982  0.80 (0.48, 1.34); 0.400  0.50 (0.05, 5.57); 0.576 |
| Unadjusted  Participant-reported only | WHO Asymptomatic  WHO Mild  WHO Moderate | 0.95 (0.27, 3.31); 0.938  0.88 (0.65, 1.19); 0.406  0.48 (0.04, 5.26); 0.545 | 1.01 (0.29, 3.05); 0.989  0.93 (0.69, 1.25); 0.645  0.50 (0.05, 5.57); 0.576 |
|  | Asymptomatic  Very mild  Mild  Moderate | 0.95 (0.27, 3.31); 0.939  0.95 (0.67, 1.34); 0.778  0.72 (0.42, 1.23); 0.229  0.48 (0.04, 5.27); 0.545 | 1.01 (0.29, 3.50); 0.989  1.01 (0.72, 1.42); 0.961  0.76 (0.45, 1.30); 0.322  0.50 (0.05, 5.58); 0.577 |
| Adjusted for site, enrolment week  All episodes | WHO Asymptomatic  WHO Mild  WHO Moderate | 1.15 (0.62, 2.14); 0.663  0.89 (0.67, 1.19); 0.439  0.42 (0.04, 4.97); 0.495 | 1.23 (0.66, 2.28); 0.517  0.93 (0.69, 1.25); 0.660  0.43 (0.05, 5.04); 0.504 |
|  | Asymptomatic  Very mild  Mild  Moderate | 1.15 (0.62, 2.14); 0.664  0.95 (0.68, 1.32); 0.744  0.76 (0.44, 1.28); 0.297  0.43 (0.04, 4.98); 0.496 | 1.23 (0.66, 2.28); 0.517  0.99 (0.71, 1.38); 0.969  0.80 (0.48, 1.35); 0.402  0.44(0.04, 5.07); 0.506 |
| Adjusted for age and sex  All episodes | WHO Asymptomatic  WHO Mild  WHO Moderate | 1.13 (0.61, 2.10); 0.698  0.86 (0.64, 1.15); 0.319  0.48 (0.04, 5.30); 0.547 | 1.20 (0.65, 2.24); 0.560  0.92 (0.69, 1.22); 0.561  0.50 (0.05, 5.58); 0.576 |
|  | Asymptomatic  Very mild  Mild  Moderate | 1.13 (0.61, 2.10); 0.698  0.91 (0.65, 1.27); 0.577  0.75 (0.45, 1.26); 0.274  0.48 (0.04, 5.29); 0.547 | 1.20 (0.65, 2.24); 0.560  0.97 (0.70, 1.35); 0.852  0.80 (0.47, 1.34); 0.387  0.50 (0.05, 5.57); 0.575 |
| Multivariable (backward selection)^4^ | WHO Asymptomatic  WHO Mild  WHO Moderate | 1.12 (0.60, 2.09); 0.729  0.82 (0.61, 1.10); 0.183  0.41 (0.03, 5.85); 0.509 | 1.23 (0.65, 2.28); 0.534  0.90 (0.66, 1.19); 0.430  0.53 (0.04, 6.90); 0.629 |
|  | Asymptomatic  Very mild  Mild  Moderate | 1.13 (0.60, 2.11); 0.711  0.87 (0.62, 1.22); 0.416  0.72 (0.43, 1.21); 0.230  0.64 (0.05, 8.24); 0.729 | 1.20 (0.64, 2.25); 0.559  0.94 (0.67, 1.31); 0.703  0.77 (0.45, 1.30); 0.328  0.71 (0.06, 9.16); 0.796 |

Abbreviations: OR=odds ratio; 95% CI= 95% confidence interval.

1. Participant with no evidence of an infection episode who completed less than 80% of the expected diary app entries were excluded because we could not be sure that they never had an infection. Participants with inconclusive infection episodes (N=36) were also removed.
2. All participants were included regardless of diary app completion, except participants with inconclusive episodes (N=36). The sensitivity analysis assumed that participant with no evidence of an infection episode who completed less than 80% of the expected diary app entries did in fact never have an infection.
3. Covariates considered as potential confounders are shown in Table S5. Covariates retained in the model were: age in years, additional number of household members, function, % work hours with patient contact, hospital department, expected to work in COVID-ward, past history of BCG vaccination, and current use of hypertension medication.
4. Covariates considered as potential confounders are shown in Table S5. Covariates retained in the model were: age in years, hospital function and department, expected to work in COVID-ward, history of BCG vaccination, and current use of hypertension medication.
5. **Cox regression models (time to first infection)**

| **BCG vs. placebo** | **Analysis population^1^**  N=1,252  241 episodes | **Sensitivity analysis population^2^**  N=1,403  241 episodes |
| --- | --- | --- |
|  | **HR (95% CI); p** | **HR (95% CI); p** |
| **Unadjusted** |  |  |
| All episodes | 0.92 (0.72, 1.19); 0.521 | 0.92 (0.72, 1.19); 0.532 |
| Participant-reported episodes only | 0.91 (0.70, 1.19); 0.493 | 0.91 (0.70, 1.19); 0.496 |
| WHO Asymptomatic episodes only | 1.19 (0.32, 4.44); 0.793 | 1.20 (0.32, 4.48); 0.783 |
| WHO Mild episodes only | 0.91 (0.70, 1.18); 0.468 | 0.91 (0.70, 1.18); 0.479 |
| Subcategory very mild episodes only | 0.96 (0.70, 1.30); 0.769 | 0.96 (0.70, 1.30); 0.785 |
| Subcategory mild episodes only | 0.76 (0.46, 1.26); 0.293 | 0.77 (0.46, 1.27); 0.302 |
| WHO Moderate episodes only | 0.48 (0.04, 5.25); 0.544 | 0.48 (0.04, 5.27); 0.546 |
| **Adjusted for site and enrolment week** |  |  |
| All episodes | 0.92 (0.71, 1.18); 0.516 | 0.92 (0.71, 1.18); 0.511 |
| Participant-reported episodes only | 0.91 (0.70, 1.18); 0.475 | 0.91 (0.70, 1.18); 0.461 |
| **Adjusted for age and sex** |  |  |
| All episodes | 0.90 (0.70, 1.16); 0.407 | 0.90 (0.70, 1.16); 0.418 |
| Participant-reported episodes only | 0.89 (0.68, 1.16); 0.386 | 0.89 (0.68, 1.16); 0.389 |
| **Multivariable (backward selection)^3^** |  |  |
| All Episodes | 0.86 (0.66, 1.11); 0.238 | 0.86 (0.67, 1.11); 0.261 |
| Participant-reported episodes only | 0.85 (0.65, 1.10); 0.218 | 0.85 (0.65, 1.11); 0.235 |

Abbreviations: HR=hazards ratio; 95% CI= 95% confidence interval.

1. Participant with no evidence of an infection episode who completed less than 80% of the expected diary app entries were excluded because we could not be sure that they never had an infection. Participants with inconclusive episodes (N=36) were also removed. Since the outcome is time to first infection, infection episodes that could not be dated dropped out of the model.
2. All participants were included regardless of diary app completion, except participants with inconclusive episodes (N=36). The sensitivity analysis assumed that participant with no evidence of an infection episode who completed less than 80% of the expected diary app entries did in fact never have an infection. Since the outcome is time to first infection, infection episodes that could not be dated dropped out of the model. An additional N=15 participants were dropped with no data regarding follow-up time.
3. Covariates considered as potential confounders are shown in Table S5. Covariates retained in the model were: age in years, additional number of household members, function, % work hours with patient contact, hospital department, expected to work in COVID-ward, past history of BCG vaccination, current use of hypertension medication.
