# Supplementary material for: BCG Vaccination of Health Care Workers Does Not Reduce SARS-CoV-2 Infections nor Infection Severity or Duration: a Randomized Placebo-Controlled Trial
Source: mBio. 2023 Mar 28;14(2):e00356-23. doi: 10.1128/mbio.00356-23 (PMC10128007; doi:10.1128/mbio.00356-23)
Supplement: TABLE S6 [file mbio.00356-23-s0010.docx]

**Table S6: Sensitivity analyses assuming inconclusive infections were or were not true infections**

1. **Logistic regression (cumulative incidence)**

| **BCG vs placebo** | | **Analysis population^1^**  N=1,309  298 episodes | **Inconclusive as no infection^2^**  N=1,344  298 episodes | **Inconclusive as infection^3^**  N=1,344  333 episodes |
| --- | --- | --- | --- | --- |
|  |  | **OR (95% CI); p** | **OR (95% CI); p** | **OR (95% CI); p** |
| **Unadjusted** | |  |  |  |
| All episodes | | 0.93 (0.72, 1.20); 0.563 | 0.92 (0.71, 1.19); 0.540 | 0.95 (0.74, 1.21); 0.661 |
| Participant-reported episodes only | | 0.89 (0.66, 1.18); 0.413 | 0.88 (0.66, 1.18); 0.396 | 0.92 (0.70, 1.20); 0.527 |
| WHO Asymptomatic episodes only | | 1.15 (0.62, 2.16); 0.654 | 1.15 (0.62, 2.16); 0.659 | 1.15 (0.62, 2.16); 0.654 |
| WHO Mild episodes only | | 0.89 (0.66, 1.18); 0.413 | 0.88 (0.66, 1.17); 0.381 | 0.89 (0.66, 1.18); 0.413 |
| Subcategory very mild episodes only | | 0.94 (0.67, 1.31); 0.715 | 0.93 (0.67, 1.30); 0.678 | 0.94 (0.67, 1.31); 0.715 |
| Subcategory mild episodes only | | 0.76 (0.45, 1.27); 0.291 | 0.75 (0.44, 1.26); 0.277 | 0.76 (0.45, 1.27); 0.291 |
| WHO Moderate episodes only | | 0.48 (0.02, 4.98); 0.545 | 0.47 (0.02, 4.94); 0.540 | 0.48 (0.02, 4.98); 0.545 |
| **Adjusted for site, enrolment week** | |  |  |  |
| All episodes | | 0.93 (0.72, 1.20); 0.577 | 0.93 (0.72, 1.20); 0.540 | 0.95 (0.74, 1.21); 0.676 |
| Participant-reported episodes only | | 0.88 (0.66, 1.18); 0.395 | 0.89 (0.66, 1.18); 0.398 | 0.91 (0.69, 1.20); 0.521 |
| **Adjusted for age and sex** | |  |  |  |
| All episodes | | 0.90 (0.70, 1.17); 0.443 | 0.90 (0.69, 1.16); 0.411 | 0.93 (0.72, 1.19); 0.559 |
| Participant-reported episodes only | | 0.86 (0.64, 1.15); 0.304 | 0.85 (0.64, 1.14); 0.283 | 0.90 (0.68, 1.18); 0.429 |
| **Multivariable (backward selection)^4^** | |  |  |  |
| All episodes | | 0.85 (0.65, 1.12); 0.249 | 0.85 (0.65, 1.11); 0.236 | 0.90 (0.70, 1.16); 0.415 |
| Participant-reported episodes only | | 0.81 (0.59, 1.09); 0.161 | 0.80 (0.59, 1.09); 0.155 | 0.87 (0.65, 1.15); 0.317 |
| **Multinomial** | |  |  |  |
| Unadjusted  All episodes | WHO Asymptomatic  WHO Mild  WHO Moderate | 1.15 (0.62, 2.14); 0.655  0.89 (0.66, 1.18); 0.413  0.48 (0.04, 5.27); 0.545 | 0.99 (0.54, 1.79); 0.966  0.88 (0.66, 1.17); 0.381  0.47 (0.04, 5.22); 0.540 | 1.15 (0.62, 2.14); 0.654  0.89 (0.66, 1.18); 0.413  0.48 (0.04, 5.27); 0.545 |
|  | Asymptomatic  Very mild  Mild  Moderate | 1.15 (0.62, 2.14); 0.655  0.94 (0.68, 1.31); 0.715  0.76 (0.45, 1.27); 0.292  0.48 (0.04, 5.28); 0.546 | 0.99 (0.54, 1.79); 0.966  0.93 (0.67, 1.30); 0.678  0.75 (0.45, 1.26); 0.277  0.47 (0.04, 5.23); 0.541 | 1.15 (0.62, 2.14); 0.654  0.94 (0.68, 1.31); 0.715  0.76 (0.45, 1.27); 0.291  0.48 (0.04, 5.26); 0.545 |
| Unadjusted  Participant-reported only | WHO Asymptomatic  WHO Mild  WHO Moderate | 0.95 (0.27, 3.31); 0.938  0.88 (0.65, 1.19); 0.406  0.48 (0.04, 5.26); 0.545 | 0.59 (0.19, 1.82); 0.358  0.87 (0.65, 1.18); 0.375  0.47 (0.04, 5.22); 0.540 | 0.95 (0.27, 3.31); 0.938  0.88 (0.65, 1.19); 0.406  0.48 (0.04, 5.27); 0.545 |
|  | Asymptomatic  Very mild  Mild  Moderate | 0.95 (0.27, 3.31); 0.939  0.95 (0.67, 1.34); 0.778  0.72 (0.42, 1.23); 0.229  0.48 (0.04, 5.27); 0.545 | 0.59 (0.19, 1.82); 0.358  0.95 (0.67, 1.33); 0.743  0.72 (0.42, 1.22); 0.218  0.47 (0.04, 5.22); 0.540 | 0.95 (0.27, 3.31); 0.938  0.95 (0.67, 1.34); 0.779  0.72 (0.42, 1.23); 0.230  0.48 (0.04, 5.26); 0.545 |
| Adjusted for site, enrolment week  All episodes | WHO Asymptomatic  WHO Mild  WHO Moderate | 1.15 (0.62, 2.14); 0.663  0.89 (0.67, 1.19); 0.439  0.42 (0.04, 4.97); 0.495 | 0.98 (0.54, 1.79); 0.956  0.89 (0.66, 1.19); 0.420  0.43 (0.04, 5.05); 0.502 | 1.15 (0.62, 2.14); 0.659  0.89 (0.67, 1.20); 0.452  0.43 (0.04, 5.09); 0.506 |
|  | Asymptomatic  Very mild  Mild  Moderate | 1.15 (0.62, 2.14); 0.664  0.95 (0.68, 1.32); 0.744  0.76 (0.44, 1.28); 0.297  0.43 (0.04, 4.98); 0.496 | 0.98 (0.54, 1.79); 0.956  0.94 (0.68, 1.31); 0.726  0.75 (0.45, 1.27); 0.287  0.43 (0.04, 5.07); 0.505 | 1.15 (0.62, 2.14); 0.659  0.95 (0.68, 1.32); 0.761  0.76 (0.45, 1.28); 0.301  0.44 (0.04, 5.11); 0.508 |
| Adjusted for age and sex  All episodes | WHO Asymptomatic  WHO Mild  WHO Moderate | 1.13 (0.61, 2.10); 0.698  0.86 (0.64, 1.15); 0.319  0.48 (0.04, 5.30); 0.547 | 0.97 (0.53, 1.76); 0.912  0.85 (0.64, 1.14); 0.284  0.47 (0.04, 5.20); 0.537 | 1.13 (0.61, 2.10); 0.702  0.86 (0.64, 1.15); 0.315  0.47 (0.04, 5.27); 0.544 |
|  | Asymptomatic  Very Mild  Mild  Moderate | 1.13 (0.61, 2.10); 0.698  0.91 (0.65, 1.27); 0.577  0.75 (0.45, 1.26); 0.274  0.48 (0.04, 5.29); 0.547 | 0.97 (0.53, 1.76); 0.913  0.90 (0.65, 1.26); 0.535  0.74 (0.44, 1.24); 0.255  0.47 (0.04, 5.20); 0.537 | 1.13 (0.61, 2.10); 0.702  0.91 (0.65, 1.27); 0.573  0.75 (0.44, 1.26); 0.271  0.47 (0.04, 5.26); 0.544 |
| Multivariable (backward selection)^5^ | WHO Asymptomatic  WHO Mild  WHO Moderate | 1.12 (0.60, 2.09); 0.729  0.82 (0.61, 1.10); 0.183  0.41 (0.03, 5.85); 0.509 | 0.97 (0.53, 1.77); 0.925  0.81 (0.60, 1.09); 0.161  0.40 (0.03, 5.80); 0.503 | 1.12 (0.60, 2.09); 0.731  0.82 (0.61, 1.10); 0.183  0.41 (0.03, 5.90); 0.510 |

|  | Asymptomatic  Very mild  Mild  Moderate | 1.13 (0.60, 2.11); 0.711  0.87 (0.62, 1.22); 0.416  0.72 (0.43, 1.21); 0.230  0.64 (0.05, 8.24); 0.729 | 0.97 (0.53, 1.78); 0.933  0.86 (0.61, 1.21); 0.384  0.72 (0.42, 1.22); 0.220  0.63 (0.05, 8.13); 0.722 | 1.13 (0.60, 2.12); 0.704  0.87 (0.62, 1.22); 0.419  0.73 (0.43, 1.23); 0.234  0.64 (0.05, 8.28); 0.731 |
| --- | --- | --- | --- | --- |

Abbreviations: OR=odds ratio; 95% CI= 95% confidence interval.

1. Participants with no evidence of an infection episode who completed less than 80% of the expected diary app entries were excluded because we could not be sure that they never had an infection. Participants with inconclusive episodes (N=36) were also removed.
2. In the randomized population, 36 episodes were coded as inconclusive, and 35 of these were in participants who completed at least 80% of expected diary app entries. These 35 were added to the analysis population as not having had an infection.
3. In the randomized population, 36 episodes were coded as inconclusive, and 35 of these were in participants who completed at least 80% of expected diary app entries. These 35 were added to the analysis population as having had an infection, with unknown severity.
4. Covariates considered as potential confounders are shown in Table S3. Covariates retained in the model were: age in years, additional number of household members, function, % work hours with patient contact, hospital department, expected to work in COVID-ward, past history of BCG vaccination, current use of hypertension medication.
5. Covariates considered as potential confounders are shown in Table S3. Covariates retained in the model were: age in years, hospital function and department, expected to work in COVID-ward, history of BCG vaccination, and current use of hypertension medication.
6. **Cox regression models (time to first infection)**

| **BCG vs placebo** | **Analysis population^1^**  N=1,252  241 episodes | **Inconclusive as no infection^2^**  N=1,287  241 episodes | **Inconclusive as infection^3^**  N=1,252  241 episodes |
| --- | --- | --- | --- |
|  | **HR (95% CI); p** | **HR (95% CI); p-value** | **HR (95% CI); p** |
| **Unadjusted** |  |  |  |
| All episodes | 0.92 (0.72, 1.19); 0.521 | 0.92 (0.71, 1.18); 0.499 | 0.92 (0.72, 1.19); 0.521 |
| Participant-reported episodes only | 0.91 (0.70, 1.19); 0.493 | 0.91 (0.70, 1.18); 0.473 | 0.91 (0.70, 1.19); 0.493 |
| WHO Asymptomatic episodes only | 1.19 (0.32, 4.44); 0.793 | 1.19 (0.32, 4.43); 0.796 | 1.19 (0.32, 4.44); 0.793 |
| WHO Mild episodes only | 0.91 (0.70, 1.18); 0.468 | 0.90 (0.69, 1.17); 0.433 | 0.91 (0.70, 1.18); 0.468 |
| Subcategory very mild episodes only | 0.96 (0.70, 1.30); 0.769 | 0.95 (0.70, 1.29); 0.731 | 0.96 (0.70, 1.30); 0.769 |
| Subcategory mild episodes only | 0.76 (0.46, 1.26); 0.293 | 0.76 (0.46, 1.25); 0.279 | 0.76 (0.46, 1.26); 0.293 |
| WHO Moderate episodes only | 0.48 (0.04, 5.25); 0.544 | 0.47 (0.04, 5.21); 0.540 | 0.48 (0.04, 5.25); 0.544 |
| **Adjusted for site, enrolment week** |  |  |  |
| All episodes | 0.92 (0.71, 1.18); 0.516 | 0.92 (0.71, 1.18); 0.510 | 0.92 (0.71, 1.18); 0.516 |
| Participant-reported episodes only | 0.91 (0.70, 1.18); 0.475 | 0.91 (0.70, 1.18); 0.477 | 0.91 (0.70, 1.18); 0.475 |
| **Adjusted for age and sex** |  |  |  |
| All episodes | 0.90 (0.70, 1.16); 0.407 | 0.89 (0.69, 1.15); 0.381 | 0.90 (0.70, 1.16); 0.407 |
| Participant-reported episodes only | 0.89 (0.68, 1.16); 0.386 | 0.89 (0.68, 1.15); 0.363 | 0.89 (0.68, 1.16); 0.386 |
| **Multivariable^4^** |  |  |  |
| All Episodes | 0.86 (0.66, 1.11); 0.238 | 0.85 (0.66, 1.10); 0.228 | 0.86 (0.66, 1.11); 0.238 |
| Participant-reported episodes only | 0.85 (0.65, 1.10); 0.218 | 0.84 (0.65, 1.10); 0.210 | 0.85 (0.65, 1.10); 0.218 |

Abbreviations: HR=hazards ratio; 95% CI= 95% confidence interval.

1. Participants with no evidence of an infection episode who completed less than 80% of the expected diary app entries were excluded because we could not be sure that they never had an infection. Participants with inconclusive episodes (N=36) were also removed. Since the outcome is time to first infection, infection episodes that could not be dated dropped out of the model.
2. In the randomized population, 36 episodes were coded as inconclusive, and 35 of these were in participants who completed at least 80% of expected diary app entries. These 35 were added to the analysis population as not having had an infection.
3. In the randomized population, 36 episodes were coded as inconclusive, and 35 of these were in participants who completed at least 80% of expected diary app entries. These 35 were added to the analysis population as having had an infection. However, they dropped out of the analysis because none of these infections could be dated.
4. Covariates considered as potential confounders are shown in Table S3. Covariates retained in the model were: age in years, additional number of household members, function, % work hours with patient contact, hospital department, expected to work in COVID-ward, past history of BCG vaccination, current use of hypertension medication.
